# Global fitting for high-accuracy multi-channel single-molecule localization

**DOI:** 10.1038/s41467-022-30719-4

**Published:** 2022-06-06

**Authors:** Yiming Li, Wei Shi, Sheng Liu, Ivana Cavka, Yu-Le Wu, Ulf Matti, Decheng Wu, Simone Koehler, Jonas Ries

**Affiliations:** 1 Department of Biomedical Engineering, Southern University of Science and Technology, Shenzhen, 518055 China; 2 European Molecular Biology Laboratory, Cell Biology and Biophysics, 69117 Heidelberg, Germany; 3 Collaboration for joint PhD degree between EMBL and Heidelberg University, Faculty of Biosciences, Heidelberg, Germany

**Keywords:** Super-resolution microscopy, Software

## Abstract

Multi-channel detection in single-molecule localization microscopy greatly increases information content for various biological applications. Here, we present globLoc, a graphics processing unit based global fitting algorithm with flexible PSF modeling and parameter sharing, to extract maximum information from multi-channel single molecule data. As signals in multi-channel data are highly correlated, globLoc links parameters such as 3D coordinates or photon counts across channels, improving localization precision and robustness. We show, both in simulations and experiments, that global fitting can substantially improve the 3D localization precision for biplane and 4Pi single-molecule localization microscopy and color assignment for ratiometric multicolor imaging.

## Abstract (web summary)

Multi-channel SMLM imaging is powerful. Here the authors report globLoc, a GPU-based global fitting algorithm, to extract maximum information from multichannel single molecule data; this gives improved localisation precision for biplane and 4Pi-SMLM and colour assignment in multi-colour astigmatic SMLM.

## Introduction

Single-molecule localization microscopy (SMLM) achieves nanometer superresolution and has become an important method for structural cell biology. Various extensions of SMLM using two or more detection channels are instrumental for this success, as they greatly increase the information content that can be extracted from samples: Multi-color SMLM imaging of proteins labeled with fluorophores of different color can probe their spatial relations and interactions. It is usually realized using two spectral channels^1–3^ or one spatial channel combined with spectral detection in a second channel^4^. Three-dimensional (3D) SMLM techniques using two or more detection channels, such as biplane^5^ or multi-plane^6^ detection, self-bending point spread functions^7^ (PSFs), supercritical-angle fluorescence detection^8,9^ and multi-phase interference^10,11^, are powerful in investigating the intrinsic 3D organization of biological structures. Two or more fluorescence polarization channels are used to probe the orientation of fluorophores^12^, offering insight into the orientation of proteins in a molecular machinery. Recently, modulation enhanced localization microscopy that uses patterned excitation with rapid detection of different phases of the pattern on multiple parts of a camera, was used to increase the resolution of SMLM by a factor of two^13–16^.

Compared to the single-channel SMLM, data analysis for all these methods is complicated by the fact that measures from two or more channels have to be combined to result in the additional information (color, *z*-position, polarization state, interference phase, etc.). Typically, this is achieved by first fitting the fluorophores individually in each channel to extract corresponding parameters, and then combining the returned parameters from different channels to obtain the extra information^1–16^. Separate fitting of an individual fluorophore present in two channels is not optimal, as we neglect the information that the fitting parameters (e.g., 3D positions and photons) are highly correlated. If instead we were to use a global fitter that links the correlated parameters across different channels, this would decrease the number of fitting parameters, improve precision and robustness of the fit and avoid ambiguity when pairing corresponding parameters. Additionally, it would allow precise analysis of a fluorophore that is very dim in one of the channels and thus would escape molecule detection when fitted separately. Despite the many benefits of analyzing separate channels simultaneously, global fitting is not widely used for the multi-channel single molecule localization. First approaches for global fitting^17–20^ lack flexibility with respect to the PSF models and fitting parameters. They are often designed for a specific imaging modality and difficult to be integrated into complete analysis workflows to be of general use. Deep-learning based SMLM data analysis software such as DECODE^21^ or deepSTORM3D^22^, have outperformed conventional fitting-based software on single-channel data, especially for dense activations beyond the single-emitter regime. However, extension to multi-channel analysis is challenging, as the complex transformations between the channels are not shift-invariant operators, and thus are difficult to be learned efficiently^23^ for a fully convolutional neuronal network.

Here, we develop globLoc, a general data analysis workflow and easy to use software for global fitting of single molecule data detected in separate channels. Its optimized analysis pipeline includes: the generation of a precise transformation among the channels, calibration of a global multi-channel PSF, a GPU based global fitter that achieves maximum accuracy (Supplementary Fig. 1, 2 and 3) and ultra-fast fitting speed (Supplementary Fig. 4), as well as post-processing routines to extract the additional information (*z*, color, interference phase, polarization, etc.). Both, in simulations and on experimental data, we show that global fitting indeed leads to a substantially improved localization precision for biplane and 4Pi-SMLM and color assignment in multi-color astigmatic SMLM.

## Results

### Workflow of globLoc

**Fig. 1:**
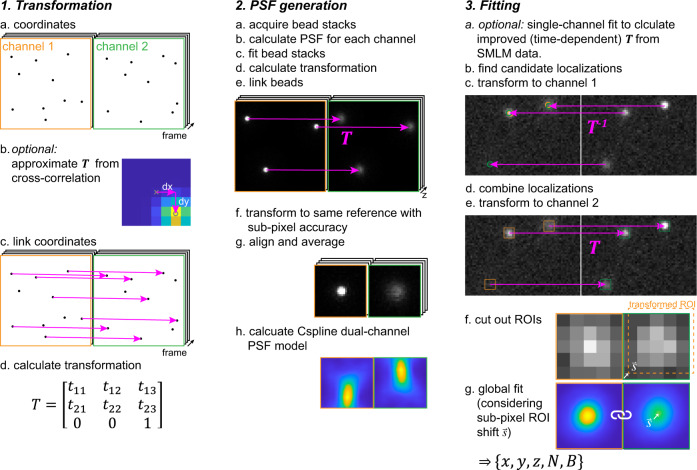
Overview of workflow of globLoc algorithm. The overall workflow of globLoc can be divided into three parts: 1. calculation of multi-channel transformation using control points from beads or single molecule data; 2. generation of multi-channel PSF models by properly averaging beads from multiple channels; 3. fitting of the multi-channel single molecule data using global maximum likelihood estimation with flexible parameter linking.

We now give an overview of the globLoc analysis workflow (Fig. 1). Details can be found in the Methods. We describe it on the example of dual-channel single molecule data. The extension to multi-channel data is straightforward. We first generate a global multi-channel experimental PSF model from image stacks of beads immobilized on a coverslip. To this end, we first calculate spline PSF models for each channel independently^24^ and fit each channel individually with the corresponding PSF model to obtain the precise bead positions. From corresponding bead positions in the two channels, we calculate the transformation between the channels. We then use cubic interpolation to register and average many bead stacks^24^, while keeping the fixed spatial relationship between the channels described by the transformation. Optionally, we re-calculate the transformation based on the actual SMLM experiment to account for channel drift. For this, we fit a sub-set of single molecule data in each channel separately using the corresponding PSF model and calculate the transformation based on the fitted coordinates. Besides using an experimental PSF model, our software also supports global fitting with a Gaussian PSF model (Supplementary Software).

After calibration of the multi-channel PSF model and the transformation between different channels, the next step of the workflow is to perform global fitting to jointly analyze the multi-channel data using maximum likelihood estimation (MLE). On a standard GPU (NVIDIA RTX3090), our implementation reached ~35,000 fits/s for regions of interest (ROI) with a size of 13×13 pixels, while the speed was ~1,000 fits/s on a CPU (Intel Core i7-8700, Supplementary Fig. 4). On simulated data for biplane SMLM, global fitting reached the Cramer-Rao-Lower-Bound (CRLB) in 3D over a large axial range (±600 nm, Supplementary Fig. 1a, b).

### Performance of globLoc on Biplane data

**Fig. 2:**
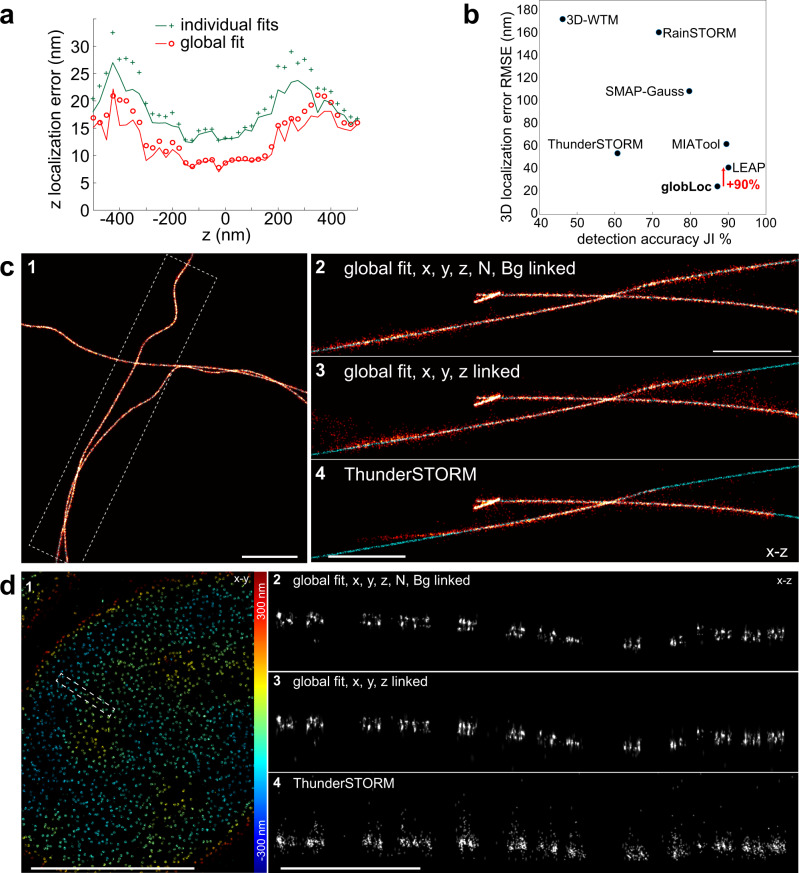
Performance of globLoc on biplane data. **a** Comparison of the *z* localization error using globLoc and using single-channel fitting followed by CRLB-weighted averaging of positions. GlobLoc improved both the minimum theoretical localization uncertainty ($\sqrt{{{\mbox{CRLB}}}}$, line) and the localization error (RMSE, circles) by approx. 1.5-fold compared to individual fitting (+, Supplementary Fig. 1). Simulations based on the biplane PSF model from the SMLM challenge 2016, 2500 detected photons in each channel and background of 20 photons per pixel. **b** Performance of different software on biplane data in the SMLM challenge 2016. GlobLoc (SMAP-global in the submission) outperformed all other software and improved the localization accuracy by 90% compared to the second-best algorithm LEAP^25^. **c** Comparison of globLoc under different parameter sharing schemes and compared to ThunderSTORM (detailed settings in Supplementary Fig. 9a) for biplane training data (MT0.N1.LD_BP) of the SMLM challenge 2016. **c2-4** Side-view cross-sections of the region as indicated by the dashed white box in c1 (c2: globLoc with *x, y, z*, photons and background photons shared; c3: globLoc with *x, y* and *z* shared; c4: ThunderSTORM.). Red-hot: fitted localizations; cyan: ground truth. **d** Comparison of globLoc on bi-plane data under different parameter sharing schemes and compared to ThunderSTORM (detailed settings in Supplementary Fig. 9b), on the example of the nuclear pore protein Nup96-AF647. **d2-4** Side-view cross-sections of the region as indicated by the dashed white box in d1 (d2: globLoc with *x*, *y*, *z*, photons and background photons linked; d3: globLoc with *x*, *y* and *z* linked; d4: ThunderSTORM.). Representative results are shown from 3 experiments for **d**. Scale bars 1 µm (c1, c2, d4), 10 µm (d1).

As globLoc is very flexible to link or unlink parameters between different channels, we compared the localization precision in the conditions of individual fit, global fit with only linking *xyz* positions and linking *xyz* positions plus photons per localization. Compared to individual fitting of the channels followed by CRLB-weighted averaging of positions^9^ (Supplementary Note 1 and 2), globLoc achieved about 1.5 times better *z* localization precision (Fig. 2a and Supplementary Fig. 1c) and more robust parameter estimation (Supplementary Fig. 1d). This resolution improvement was further confirmed by participating in the continuously running 2016 SMLM Software Challenge^25^, in which globLoc improved the 3D localization precision by almost a factor of two on biplane data, compared to the second-best performing algorithm LEAP (Fig. 2b). Our own comparison on the training data set (simulated microtubules) showed a clear improvement compared to the popular SMLM analysis software ThunderSTORM^26^ (Fig. 2c). The improvement of globLoc compared to ThunderSTORM was even more apparent when we analyzed experimental SMLM data of nuclear pore complex (NPC) protein Nup96, which we used as a reference standard^27^. In contrast to ThunderSTORM, globLoc was able to clearly resolve the two-ring structure of the NPC (Fig. 2d). This is likely not only due to a better localization precision, but also an improved robustness of the fit.

### Performance of globLoc on 4Pi-SMLM data

**Fig. 3:**
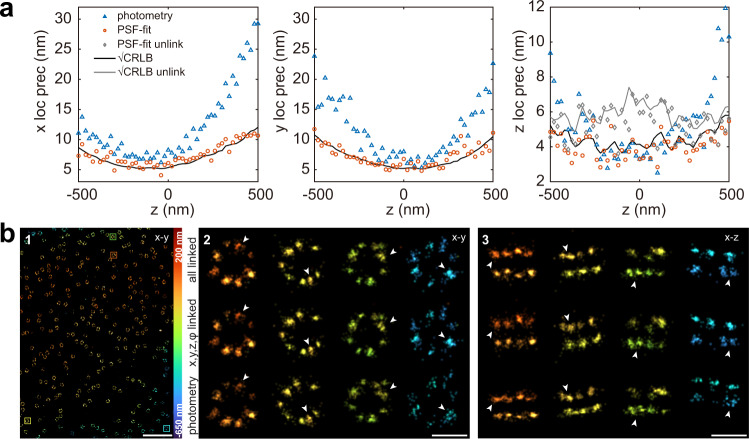
Performance of globLoc on 4Pi-SMLM data. **a** Localization precision for 4Pi-SMLM imaging of fluorescent beads. A single 40 nm red fluorescence bead (F8793, Invitrogen) was imaged at *z* positions from −500 nm to 500 nm with 20 nm step size and 37 frames were collected at each *z* position. The estimated photons/objective is 464 ± 40 (mean ± s.t.d) and the background photons/pixel is 0.47 ± 0.04 (mean ± s.t.d). The localization precision is calculated as the standard deviation. Theoretical estimated precision ($\sqrt{{{\mbox{CRLB}}}}$) in *z* increases when unlinking the photon and background in four phase channels. **b** Comparison of globLoc on 4Pi-SMLM data under different parameter sharing schemes and compared to the state-of-the art analysis (photometry)^11^, on the example of Nup96-AF647. **b2**–**3** Top-view and side-view reconstructions of selected NPCs at different *z* positions as indicated in b1 (all linked: globLoc with *x*, *y*, *z*, photons and background photons linked; *x,y,z*, $\varphi$ linked: globLoc with *x*, *y* and *z* and the phase linked). Representative results are shown from 3 experiments for (**b**). Scale bars 10 µm 1 µm (b1), 100 nm (b2-3).

GlobLoc is not limited to 2 channels. We implemented four-channel fitting for 4Pi-SMLM with multi-phase interference, using an advanced experimental 4Pi-PSF model that we developed recently^28^ (Supplementary Note 3). By fitting all four phase images globally with such a spline-interpolated experimental PSF model, globLoc achieved the CRLB in all dimensions, and greatly improved precision as well as accuracy compared to the state-of-the-art analysis (Fig. 3a and Supplementary Fig. 3). As for biplane data, we found that additionally linking the photon number between different channels improved the localization precision in *z* by up to 1.5 times compared to only linking *xyz* (Supplementary Fig. 3). We also demonstrated the resolution improvement with experimental 4Pi-SMLM data of Nup96. The clusters, which represent the eight corners of the Nup96 within the nuclear pore complex, reconstructed by globLoc with all parameters linked are smaller than those from photometry and globLoc without linking photons (Fig. 3b).

### Performance of globLoc on ratiometric multicolor SMLM data

**Fig. 4:**
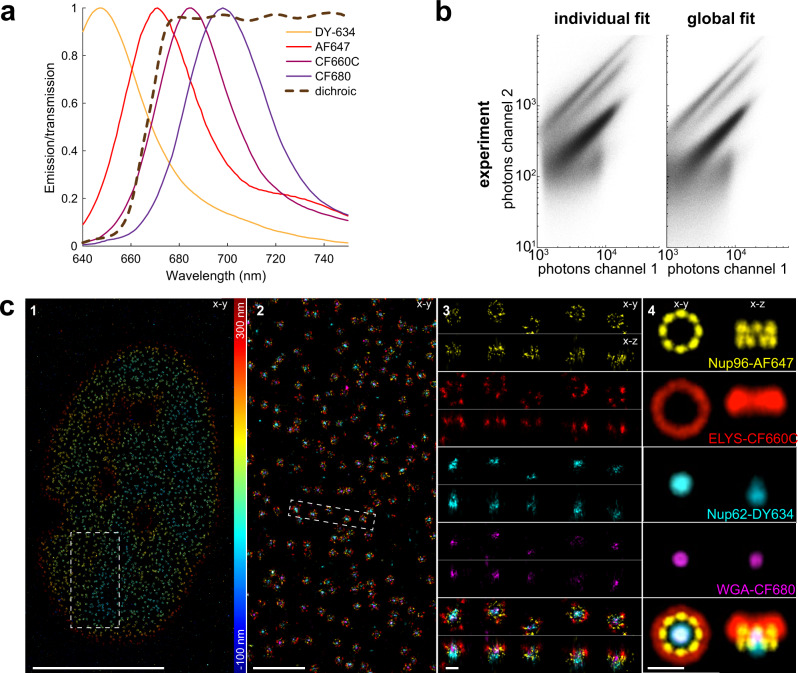
Performance of globLoc on ratiometric multi-color data. **a** Emission spectra of DY-634, AF647, CF660C and CF680 and transmission profile of the dichroic beamsplitter. The experimentally determined ratio of photons between dark and bright channels for these four dyes are 0.39, 0.21, 0.07 and 0.02. **b** Scatter plot of the fitted transmitted versus reflected photons per localization in a typical 4 color experiment using global fit and individual fit separately. **c** 4 color 3D imaging of Nup62-DY634, Nup96-AF647, ELYS-CF660C and WGA-CF680 in the NPC using globLoc. **c1**, Top view of bottom nuclear membrane. **c2**, Zoomed image of the region indicated in d1. **c3**, Top view and side view images of the region indicated in c2. **c4**, Average of 200 NPC images by registering the Nup96 structures that we used as a reference. See Supplementary Movie 1. Representative results are shown from 3 experiments for (**c**). Scale bars 10 µm (**c1**), 1 µm (**c2**), 100 nm (**c3**-**4**).

Ratiometric multicolor SMLM images two or more dyes with overlapping emission spectra in two spectral channels and assigns the color of single molecules based on the relative number of photons detected in each channel (Fig. 4a and Supplementary Fig. 5a). It has many advantages over conventional multicolor superresolution imaging using dyes with well separated emission spectra^1,3,29^: (1) it has a negligible channel shift and chromatic aberration; (2) many of the best “blinking” dyes have similar emission spectra in the dark red range and are compatible with similar imaging conditions; (3) multi-color imaging can be performed simultaneously with one excitation laser. A key challenge for ratiometric color assignment is to precisely determine the photon number of the single molecules to distinguish their color. By using salvaged fluorescence reflected by the main excitation dichroic mirror, Zhang et al. have shown 3 color superresolution imaging of biological structures in 3D at 5–10 nm localization precision using 4Pi-SMS microscopy^3^. However, the salvaged fluorescence was only used for color assignment and did not contribute to the molecule localization.

Global fitting with globLoc improves the accuracy of determining the photons per localization (Supplementary Fig. 2c) and thus the color assignment in both simulation and experiment (Fig. 4b, Supplementary Figs. 5, 6 and 7), while utilizing all detected photons for localization. To exploit our finding that linking photon numbers across channels increases the accuracy, we implemented a fitting approach in which globLoc fixes the relative photon numbers across the channels to different pre-calculated values and chooses the solution with the maximum likelihood (Supplementary Fig. 6). This approach reduced crosstalk during color assignment and minimized rejection of single molecules with close intensity ratios. It also makes a post-processing step for color assignment obsolete (Supplementary Fig. 7).

These innovations of globLoc enabled us to image and faithfully distinguish a record of 4 colors simultaneously in ratiometric 3D SMLM (Fig. 4c, Supplementary Fig. 8 and Supplementary Video 1) and image Nup96, Nup62, Elys and WGA within single NPCs labeled with the dyes AF647, DY634, CF660C and CF680 with no apparent cross-talk. We averaged 200 NPC images by registering the Nup96 structures that we used as a reference^30^. This protein density map shows the average positions of the four NPC proteins, with Nup96 forming two rings with an 8-fold symmetry, Elys forming a large ring and Nup62 and WGA localizing at the central channel of the pore. It is worth noting that these are the average distributions of the fluorophores, which can be different from the distribution of the epitopes due to linkage errors introduced by the size of the antibodies and their non-random orientations.

**Fig. 5:**
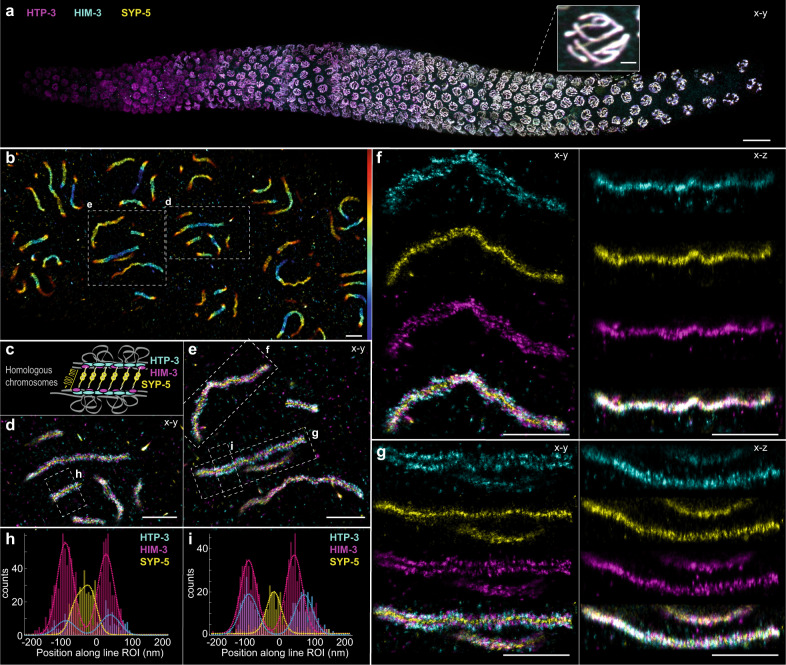
Three-color 3D imaging of the synaptonemal complex in *C. elegans*. Ratiometric three color 3D astigmatism imaging of the synaptonemal complex components HTP-3, HIM-3 and the N-terminus of SYP-5. **a** Three channel maximum intensity projection image of a whole mount *C. elegans* gonad showing the synaptonemal complex components HTP-3, HIM-3, and SYP-5. To create the image of the whole gonad, individual tiles were stitched using the Grid/Collection stitching Fiji plugin^45^. **b** Top view of the bottom-most section of meiotic nuclei, with rendered SMLM localizations of the synaptonemal complex components colored by their *z* coordinate (z range −400 to 400 nm). **c** Cartoon showing relative positioning of the target proteins within the structure of the synaptonemal complex (based on previous research^31–33^). **d**, **e** Zoomed-in image of the regions indicated in (**b**). **f**, **g** Top-view and side-view images of the synaptonemal complex stretches indicated in (**e**). **h**, **i** Histograms of localization counts along the 300 nm wide ROIs, as indicated in (**d**, **e**). Double Gaussian fits for localization distributions of individual proteins resulted in the following distances: HTP-3: 124 nm, HIM-3: 114 nm, SYP-5: 37 nm (**h**) and HTP-3: 157 nm, HIM-3: 130 nm, SYP-5: 0 nm (**i**). Representative results are shown from 2 experiments. Scale bars 10 μm (**a**), 1 μm (**a** inset, **b**, **d**–**g**).

To demonstrate the performance of globLoc on an especially challenging sample, we performed ratiometric three color 3D imaging of the synaptonemal complex in *C. elegans* (Fig. 5a). Individual synaptonemal complexes could be clearly resolved in 3D (Fig. 5b). Three different components of the synaptonemal complex, HTP-3, HIM-3 and the N-terminus of SYP-5, were well separated without visible cross-talk. The spatial arrangement of these 3 components were in well agreement with previous research^31–33^ (Fig. 5c–f).

Taken together, we demonstrated the potential of globLoc on the challenging experiments such as 3 color and 4 color 3D super resolution imaging of various biological samples.

## Discussion

To summarize, we demonstrated that linking shared parameters during multi-channel single molecule localization substantially improves localization accuracy and reduces color assignment crosstalk. globLoc not only improves the localization accuracy, but also increases the robustness of the fit compared to fitting multiple channels individually (Supplementary Fig. 1d and Supplementary Fig. 2d). This is important, as the information is split into different channels and the signal to noise ratio (SNR) is poorer in each individual channel, which results in a large error in the parameter estimation. As a result, globLoc could precisely reconstruct biplane and 4Pi-SMLM data over a large axial range and faithfully distinguished a record of ratiometric 4 color data imaged simultaneously.

Deep learning based SMLM analysis has been shown to greatly improve precision, especially for imaging data with a high density of single molecules^21,22^. However, these methods rely on well-trained networks, which need to be retrained for different imaging conditions, such as different SNR. For multi-channel data analysis, the situation is much more complex as different parameter linking schemes need be exploited for different imaging modalities (e.g., biplane, ratiometric multicolor). Furthermore, incorporating a shift-variant channel transformation operator to the convolutional neuron network (CNN) is still challenging as conventional CNN cannot learn the spatial information efficiently. In contrast, globloc is very convenient in terms of parameters sharing and flexible PSF modeling. It is capable to analyze different multi-channel data by easily incorporating the transformation function in the fitter. Therefore, deep learning-based algorithms are well suited for single-channel high-density data, whereas globLoc will be the method of choice for multi-channel data analysis of standard emitter densities.

In the continuously running SMLM Challenge^25^, globLoc improved the localization precision by almost a factor of 2 compared to the second-best software, showcasing the limited performance of current analysis software for multi-channel data. To allow anyone to directly and easily use the full functionality of globLoc (complete multi-channel calibration pipeline, versatile PSF model, flexible parameter sharing and fast fitting speed accelerated by GPU), we fully integrated it in SMAP^34^. In addition, we provide open-source example code in MATLAB and Python to allow simple and direct integration in custom software. We believe that globLoc will enable many groups to substantially improve their analysis workflows for multi-channel SMLM.

## Methods

### Calculation of multi-channel transformation

Global fitting of multi-channel data relies on knowing the precise transformation among the channels. We developed a routine to calculate transformations from coordinates (bead positions or positions of single fluorophores) that we used during the generation of the multi-channel PSF model and for global fitting of multi-channel single molecule data. We describe our algorithm for a two-channel transformation (reference ${\vec{x}}_{r}$ and target channel ${\vec{x}}_{t}$). A multi-channel transformation is represented as several two-channel transformations from all target channels to the same reference channel. Our algorithm is as follows (Fig. 1):

1. Obtain approximate transformation ${{{{{{\boldsymbol{T}}}}}}}_{0}$. This can be the transformation calculated in a previous experiment. Alternatively, we calculate it by first binning the coordinates in super pixels with a size of 50 nm. Then, we calculate the image cross-correlation and determine the position of the cross-correlation peak with sub-pixel accuracy from the position of the brightest pixel in a 4-fold upscaled image calculated by Gaussian filtering followed by cubic interpolation. The position of the cross-correlation peak corresponds to the shift between the channels, which we then use as ${{{{{{\boldsymbol{T}}}}}}}_{0}$.
2. We transform all target coordinates to the reference channel using ${{{{{{\boldsymbol{T}}}}}}}_{0}$: ${\vec{x}}_{t}^{{\prime} }={{{{{{\boldsymbol{T}}}}}}}_{0}^{-1}{\vec{x}}_{t}$.
3. We link coordinates in the reference and target channel if they are closer than a maximum distance $\rho$: $\left|{\vec{x}}_{t}^{{\prime} }-{\vec{x}}_{r}\right| \, < \, \rho$. For fluorophores from SMLM experiments we only link coordinates from the same frame.
4. We calculate the precise transformation ${{{{{\boldsymbol{T}}}}}}$ based on the linked ${\vec{x}}_{r}$ and ${\vec{x}}_{t}$ as anchor points. Usually, we use a projective transformation where ${{{{{\boldsymbol{T}}}}}}$ is represented by a $3\times 3$ matrix, but we can use all transformations supported by Matlab, e.g., polynomial transformations.
5. If necessary, we repeat steps 3 and 4 with reduced $\rho$.

### Generation of multi-channel PSF models

Our algorithm to generate multi-channel PSF models from bead stacks is an extension of our work on generating single-channel experimental PSF models^24^. Again, we illustrate the steps of our algorithm on the dual-channel example, an extension to N channels is straight forward (Fig. 1):

1. We find candidate bead positions in each channel by calculating the mean image over all *z*-positions, Gaussian filtering and finding of local maxima above a user-defined threshold. These candidate positions are integers in the unit of camera pixels.
2. If no transformation among the channels exists, we first generate single-channel PSF models for each channel separately. We then fit the bead images using these new PSF models and finally use the fitted localizations to calculate ${{{{{\boldsymbol{T}}}}}}$ as described above.
3. We transform the coordinates of the candidate bead positions from the reference to the target channel: ${\vec{x}}_{r}^{{\prime} }={{{{{\boldsymbol{T}}}}}}{\vec{x}}_{r}$. These target coordinates are continuous coordinates; thus we calculate the nearest integer pixel position by rounding the transformed coordinates $\left\lfloor {\vec{x}}_{r}^{{\prime} }\right\rfloor$and calculating the shift between the rounded and original transformed coordinates $\vec{s}={\vec{x}}_{r}^{{\prime} }-\left\lfloor {\vec{x}}_{r}^{{\prime} }\right\rfloor$.
4. We cut out ROIs around ${\vec{x}}_{r}$ and $\left\lfloor {\vec{x}}_{r}^{{\prime} }\right\rfloor$ out of the bead stacks and shift the ROIs of the target channel by $\vec{s}$ using cubic interpolation. If the target channel is mirrored with respect to the reference channel, we mirror the target ROI. This ensures that beads in both channels are shifted in the same direction during registration. Finally, we concatenate image stacks in both channels to form a single 3D array.
5. We create an initial template by averaging the 3D arrays over all beads and use 3D cross-correlation to register all beads to this template.
6. We reject those beads that have an insufficient overlap with the template (quality control) and calculate the next template as the average of the remaining shifted beads. We then register the central part of each bead to the new template.
7. We normalize the beads by the sum of the central slice of the reference stack.
8. We slightly filter the PSF models in *z* with a smoothing bspline and calculate a cspline representation for each channel.
9. To validate the PSF calibration, we fit each bead in the bead stack and compare the fitted *z* position with the true *z* position as denoted by the frame in the image stack.

### Extraction of multi-channel single molecule data

We implemented the workflow for global fitting of single molecule blinking events in the following way (again illustrated for two channels):

1. We calculate the global PSF model as described above from bead stacks.
2. Optionally, especially if we did not acquire bead stacks on the same day as the SMLM measurements, we calculate an improved transformation by fitting single molecule localizations in each channel independently and then using these localizations as anchor points to calculate ${{{{{\boldsymbol{T}}}}}}$ as described above. Otherwise, we use the transformation from the bead calibration.
3. We find candidate peaks in all channels using a difference of Gaussian filter and maximum finding. We then transform all candidates back to the first channel and average close-by candidate positions to obtain the coordinates of the candidates in the reference channel. Finally, we round to the nearest integer pixels to obtain ${\vec{x}}_{r}$.
4. As described for the beads, we transform the candidate positions to the target channels ${\vec{x}}_{r}^{{\prime} }={{{{{\boldsymbol{T}}}}}}{\vec{x}}_{r}$ and calculate the shift between the rounded and original transformed coordinates $\vec{s}={\vec{x}}_{r}^{{\prime} }-\left\lfloor {\vec{x}}_{r}^{{\prime} }\right\rfloor$.
5. Then we cut out ROIs around ${\vec{x}}_{r}$ and $\left\lfloor {\vec{x}}_{r}^{{\prime} }\right\rfloor$. If the two channels are mirrored, we additionally mirror the ROIs and $\vec{s}$.

### Maximum likelihood estimation of multi-channel single molecule data

We use a maximum likelihood estimator that jointly optimizes the combined likelihood across different channels. The objective function for MLE across different channels is given by:

1 $${\chi }_{{mle}}^{2}=2\left(\mathop{\sum}\limits_{i}\mathop{\sum}\limits_{k}\left({\mu }_{{ki}}-{M}_{{ki}}\right)-\mathop{\sum}\limits_{i}\mathop{\sum}\limits_{k,{M}_{{ki}} > 0}{M}_{{ki}}\,{{{{{\rm{ln}}}}}}\left(\frac{{\mu }_{{ki}}}{{M}_{{ki}}}\right)\right).$$

Here, ${M}_{{ki}}$ is the measured photon number in the *k*th pixel of the *i*th channel. ${\mu }_{{ki}}$ is the expected photon number in the *k*th pixel of the *i*th channel. Similar to previous implementations^24,35,36^, we used a modified Levenberg-Marquardt (L-M) algorithm to minimize ${\chi }_{{mle}}^{2}$ (Supplementary Note 4). For the multichannel nonlinear optimization process, the parameters can be classified as either shared (global) or non-shared (local) parameters, ($\theta \in {{{{{{\boldsymbol{(}}}}}}{{{{{\boldsymbol{\theta }}}}}}}_{{{{{{\boldsymbol{p}}}}}}}{{{{{\boldsymbol{,}}}}}}\,{{{{{{\boldsymbol{\theta }}}}}}}_{{{{{{\boldsymbol{qi}}}}}}}$). Here, ${{{{{{\boldsymbol{\theta }}}}}}}_{{{{{{\boldsymbol{p}}}}}}}$ is the set of global parameters and ${{{{{{\boldsymbol{\theta }}}}}}}_{{{{{{\boldsymbol{qi}}}}}}}$ is the set of local parameters of *i*th channel. The global parameters appear in all channels while the local parameters appear only in the individual channel. Depending on the imaging modality, any fitting parameter $\theta$ (*x*, *y*, *z/*${\sigma }_{{PSF}}$, photons, background) can be either linked as a global parameter among the channels or treated as a local parameter with different values in each channel. For global parameters, we define a transformation function to link parameters of different channels (translation and scale). The shared parameter in the *i*th channel can be written as: ${\theta }_{{pi}}={S}_{{pi}}{\theta }_{p}+\triangle {\theta }_{pi}.$ Here, ${S}_{{pi}}$ and $\triangle {\theta }_{pi}$ are the scaling and translation factor, respectively. In this work, $\triangle {\theta }_{xi}$ and $\triangle {\theta }_{yi}$ are defined as the shifts $\vec{s}$ between the transformed ROI position and the actual ROI position which is rounded to integer pixels, as defined in item 4 of the section: Extraction of multi-channel single molecule data. The ratio of the photons between different channels ${S}_{{Ni}}$, used for fixed photon ratio fitting, is determined from experimental single molecule data as the mean of the detected photons per localization for each dye. Therefore, the first derivative for a global parameter ${\theta }_{pi}$ in the *i*th channel is given by:

2 $$\frac{\partial {u}_{{ki}}}{\partial {\theta }_{pi}}={S}_{{pi}}\frac{\partial {u}_{{ki}}}{\partial {\theta }_{p}}.$$

The first derivative for a local parameter ${\theta }_{{qj}}$ in the *i*th channel is 0 when *i* is not equal to *j*:

3 $$\frac{\partial {u}_{{ki}}}{\partial {\theta }_{{qj}}}=0,\,{if}\,i\,\ne\, j.$$

Therefore, the Jacobian matrix can be defined as:

4 $${J}_{m}={\sum }_{i}{\sum }_{k}\frac{\partial {\chi }_{{mle}}^{2}}{\partial {\theta }_{{mi}}}=\left\{\begin{array}{c}{\sum }_{i}{\sum }_{k}{S}_{{mi}}\frac{\partial {u}_{{ki}}}{\partial {\theta }_{{mp}}}\frac{({M}_{{ki}}-{u}_{{ki}})}{{u}_{{ki}}},{\theta }_{{mi}}\in {{{{{{\boldsymbol{\theta }}}}}}}_{{{{{{\boldsymbol{p}}}}}}}\\ {\sum }_{k}\frac{\partial {u}_{{ki}}}{\partial {\theta }_{{mi}}}\frac{({M}_{{ki}}-{u}_{{ki}})}{{u}_{{ki}}},{\theta }_{{mi}}\in {{{{{{\boldsymbol{\theta }}}}}}}_{{{{{{\boldsymbol{qi}}}}}}}\end{array}\right..$$

The Hessian matrix is defined as:

5 $${H}_{m,n}={\sum }_{i}{\sum }_{k}\frac{\partial {\chi }_{{mle}}^{2}}{\partial {\theta }_{{mi}}}\frac{\partial {\chi }_{{mle}}^{2}}{\partial {\theta }_{{ni}}}=\left\{\begin{array}{c}{\sum }_{i}{\sum }_{k}{S}_{{mi}}\frac{\partial {u}_{{ki}}}{\partial {\theta }_{{mp}}}{S}_{{ni}}\frac{\partial {u}_{{ki}}}{\partial {\theta }_{{np}}}\frac{{M}_{{ki}}}{{{u}_{{ki}}}^{2}},{\theta }_{{mi}}\in {{{{{{\boldsymbol{\theta }}}}}}}_{{{{{{\boldsymbol{p}}}}}}}{{{{{\boldsymbol{,}}}}}}\,{\theta }_{{ni}}\in {{{{{{\boldsymbol{\theta }}}}}}}_{{{{{{\boldsymbol{p}}}}}}}\\ {\sum }_{k}{S}_{{mi}}\frac{\partial {u}_{{ki}}}{\partial {\theta }_{{mp}}}\frac{\partial {u}_{{ki}}}{\partial {\theta }_{{ni}}}\frac{{M}_{{ki}}}{{{u}_{{ki}}}^{2}},{\theta }_{{mi}}\in {{{{{{\boldsymbol{\theta }}}}}}}_{{{{{{\boldsymbol{p}}}}}}}{{{{{\boldsymbol{,}}}}}}\,{\theta }_{{ni}}\in {{{{{{\boldsymbol{\theta }}}}}}}_{{{{{{\boldsymbol{q}}}}}}{{{{{\boldsymbol{i}}}}}}}\\ {\sum }_{k}\frac{\partial {u}_{{ki}}}{\partial {\theta }_{{mi}}}\frac{\partial {u}_{{ki}}}{\partial {\theta }_{{ni}}}\frac{{M}_{{ki}}}{{{u}_{ki}}^{2}},{\theta }_{{mi}}\in {{{{{{\boldsymbol{\theta }}}}}}}_{{{{{{\boldsymbol{qi}}}}}}}{{{{{\boldsymbol{,}}}}}}\,{\theta }_{{ni}}\in {{{{{{\boldsymbol{\theta }}}}}}}_{{{{{{\boldsymbol{q}}}}}}{{{{{\boldsymbol{i}}}}}}}{{{{{\boldsymbol{,}}}}}}\,m=n\\ 0,{\theta }_{{mi}}\in {{{{{{\boldsymbol{\theta }}}}}}}_{{{{{{\boldsymbol{qi}}}}}}}{{{{{\boldsymbol{,}}}}}}\,{\theta }_{{ni}}\in {{{{{{\boldsymbol{\theta }}}}}}}_{{{{{{\boldsymbol{q}}}}}}{{{{{\boldsymbol{i}}}}}}}{{{{{\boldsymbol{,}}}}}}\,m \,\ne\, n\end{array}\right..$$

In the L-M algorithm, we updated the parameters by solving the linear equations: $({{{{{\boldsymbol{H}}}}}}+\lambda {{{{{\boldsymbol{I}}}}}})\triangle {{{{{\boldsymbol{\theta }}}}}}={{{{{\boldsymbol{J}}}}}}$, with $\lambda$ the damping factor and ***I*** a diagonal matrix equal to the diagonal elements of the Hessian matrix. The detailed algorithm can be found in Supplementary Note 4. Depending on different fitting modalities, we then calculate parameters of interest (e.g., color, polarization or z-position) from the fitted parameters (e.g., number of photons in each channel). Finally, we perform the usual post processing steps such as merging of localizations persisting over consecutive frames, drift correction and filtering based on log-likelihood and localization precision.

### Simulation and analysis of multi-channel data with experimental PSF models

For biplane data simulation (Fig. 2a and Supplementary Fig. 1), the biplane experimental PSF model of the SMLM challenge 2016 was used. For each single molecule, we use 5000 detected photons and 40 background photons for the simulation. The photons were then split into two channels with 1:1 ratio. 1000 single molecule images were simulated for each *z* position (range from −600 to 600 nm) on a ROI of 15 × 15 pixels. Only Poisson noise was added to the images. The simulated biplane single molecule data was then fitted with three different schemes: (1) global fit with *x*, *y*, *z*, photons and background photons shared; (2) global fit with *x*, *y*, and *z* parameters shared; (3) individual fit for each channel and combination of the parameters of different channels with CRLB weighted arithmetic mean (Supplementary Note 1). The localization accuracy was calculated as the *root mean square error* (RMSE) of the fitted coordinates compared to the ground truth.

For the ratiometric astigmatic simulation (Supplementary Fig. 2, Supplementary Fig. 5 and Supplementary Fig. 6), a dual channel astigmatic experimental PSF acquired from multicolor beads was used. The photon distribution of these 4 different dyes were used for simulation: DY634, AF647, CF660C and CF680. The ratio of photons between two channels for the different dyes was determined from experimental data corresponding to Fig. 4c as the mean of the detected photons per localization for each dye. We found photon ratios of ${I}_{2}/{I}_{1}=$ 0.39, 0.21, 0.07 and 0.02 for DY634, AF647, CF660C and CF680, respectively. Here, ${I}_{1}$ and ${I}_{2}$ are the photons from the bright and dark channels, respectively. For comparison of localization accuracy and CRLB (Supplementary Fig. 2), the photon ratio of 0.25 was used. 1,000 molecules with a ROI size of 15 × 15 pixels at each *z* position were used to calculate the RMSE. For ratiometric color separation (Supplementary Fig. 5 and Supplementary Fig. 6), 50,000 single molecules were randomly placed at axial positions between −600 and 600 nm. The photon distribution of each dye follows the distribution of the experimentally acquired single molecules (Supplementary Fig. 6a). Three different methods were used to determine the color information: (1) The dual channel data was fitted separately; (2) The dual channel data was fitted globally with x, y and z as global parameters, photons and background as local parameters; (3) The dual channel data was fitted globally with x, y, z and photons as global parameters, background photons as shared parameter. The ratio of the photons between different channels was fixed during fit. For the first two methods, the color discrimination was realized by thresholding the normalized photon ratio: $({I}_{1}-{I}_{2})/({I}_{1}+{I}_{2})$. For the third method, the dual channel data was fitted with different fixed and pre-determined photon ratios of all 4 dyes between the two channels and we then chose the solution with the maximum likelihood.

For 4Pi single molecule data simulation (Supplementary Fig. 3), 2000 photons/localization and 20 background photons/pixel were used for each objective. A full vectorial PSF model^37^ was used for simulations with the following parameters: NA 1.35, refractive index 1.40 (immersion medium and sample) and 1.518 (cover glass), emission wavelength 668 nm, astigmatism 100 mλ. 1000 4Pi single molecule images with a ROI size of 15 × 15 pixels were simulated at each *z* position with four phase channels (0, π/2, π, 3π/2). The *x* and *y* positions are randomly distributed within −1 to 1 pixels around the center of each ROI. The simulated 4Pi single molecule data was then fitted with three different approaches: (1) global fit using IAB-based 4Pi-PSF model with *x*, *y*, *z*, phase, photons and background photons shared; (2) global fit using IAB-based 4Pi-PSF model with *x*, *y*, z and phase, parameters shared; (3) photometry based methods^11^.

### GPU implementation of globLoc and speed evaluation

We implemented the globLoc fitter with both spline and pixelated Gaussian PSF model^38^ using CUDA C/C++ in NVIDIA CUDA®-enabled graphic cards. The framework of the L-M iterative fitting method follows the previous work^24^. Each thread is pointed to a multi-channel single-molecule data and performs the entire fitting process for each single molecule. We put the single-molecule data in the global memory of the GPU and employed 64 threads for each block for the computation. Both the CPU and GPU based C++ code were compiled in Microsoft Visual Studio 2019 and called via Matlab 2019a (Mathworks) MEX files. For speed evaluation (Supplementary Fig. 4), we ran the CPU code on a personal computer using an Intel Core i7-8700 processor clocked at 3.2 GHz with 16GB memory. For the GPU-based evaluation, an NIVDA GeForce GTX 3090 graphics card with 24.0 GB memory was used.

### State-of-the-art workflows used for comparison

For biplane data analysis, we compared globLoc with the widely used ThunderSTORM software^26^. In the ThunderSTORM biplane analysis pipeline, a homography transformation is constructed from paired coordinates of the two channels. The biplane data is then fitted simultaneously using an astigmatic Gaussian PSF model. The detailed parameters used are shown in Supplementary Fig. 9 and Supplementary Table 1. For ratiometric multi-color assignment, we also compared to a workflow similar to that presented by Lehann et al (Supplementary Fig. 8)^29^. In short, localizations are fitted separately in the two channels. We then construct the transformation as described above and associate corresponding localizations in the two channels. Color assignment is then based on the relative fitted photon numbers.

### Cell culture

Before seeding of cells, high-precision 24 mm round glass coverslips (No. 1.5H, catalog no. 117640, Marienfeld) were cleaned by placing them overnight in a methanol:hydrochloric acid (50:50) mixture while stirring. After that, the coverslips were repeatedly rinsed with water until they reached a neutral pH. They were then placed overnight into a laminar flow cell culture hood to dry them before finally irradiating the coverslips by ultraviolet light for 30 min.

Cells were seeded on clean glass coverslips 2 days before fixation to reach a confluency of about 50 – 70% on the day of fixation. They were grown in growth medium (DMEM (catalog no. 11880-02, Gibco)) containing 1× MEM NEAA (catalog no. 11140-035, Gibco), 1× GlutaMAX (catalog no. 35050-038, Gibco) and 10% (v/v) fetal bovine serum (catalog no. 10270-106, Gibco) for approximately 2 days at 37 °C and 5% CO_2_. Before further processing, the growth medium was aspirated, and samples were rinsed with PBS (RT) to remove dead cells and debris. Unless otherwise stated, all experimental replicates were performed on cells of a different passage with separated sample preparation.

### Imaging Buffer

Glucose oxidase/catalase buffer supplemented with cysteamine (MEA) was used to image Nup96-SNAP-AF647-ELYS-CF660C-Nup62-DY634-WGA-CF680. GLOX+MEA contained 50 mM Tris/HCl pH8, 10 mM NaCl, 10% (w/v) D-glucose, 500 µg/ml glucose oxidase, 40 µg/ml glucose catalase and 35 mM MEA in H_2_O.

### Preparation of four-color NPC samples

Cells (Nup96-SNAP-tag, catalog no. 300444, CLS Cell Line Service, Eppelheim, Germany) on glass coverslips were prefixed in 2.4% (w/v) Formaldehyde (FA, 28906, ThermoFischer Scientific) in PBS for 20 s before incubating them 10 min in 0.5% (v/v) Triton X-100 in PBS. Fixation was completed in 2.4% (w/v) FA in PBS for 20 min. FA was quenched for 5 min in 100 mM NH_4_Cl in PBS and then washed 3 × 5 min in PBS. Fixed cells were blocked with Image-IT signal enhancer for 30 min and then incubated with 1 µM BG-AF647(#S9136S, New England Biolabs), 0.5% BSA and 1 mM DTT(Dithiothreitol) in PBS for 1 h to stain Nup96-SNAP-tag. Cells were washed 3x for 5 min with PBS and subsequently blocked with 5% (v/v) NGS (normal goat serum, catalog no. PCN5000, lifeTech) in PBS for 1 h. Primary antibody labeling against ELYS was achieved by incubation with rabbit anti-AHCTF1 primary antibody(HPA031658, Sigma-Aldrich) diluted 1:40 in 5% (v/v) NGS in PBS for 1 h. Coverslips were washed 3 times for 5 min with PBS to remove unbound antibody and subsequently stained with CF660C labeled goat anti-rabbit antibody (20183, Biotium, Fremont, CA) diluted 1:150 in PBS containing 5% (v/v) NGS for 1 h. After 3 washes with PBS for 5 min, the sample was postfixed for 30 min using 2.4% (w/v) FA in PBS, rinsed with PBS, quenched in 50 mM NH_4_Cl for 5 min and rinsed 3 × 5 min with PBS. Labeling against Nup62 was performed by incubation with mouse anti-Nup-62 primary antibody (610498, BD Bioscience) diluted 1:50 in 5% NGS/PBS for 2 h, 3x 5 min washes of the coverslips with PBS and incubation over night at 4degC with 1:150 diluted secondary donkey anti-mouse-DY634 antibody in 5%NGS/PBS. Unbound antibody was removed from the sample by washing 5 times with PBS. All incubations except otherwise stated were carried out at RT. Buffers used were also pre-equilibrated to RT.

Shortly before imaging, the sample was incubated for 10 min with 1:5000 diluted WGA-CF680 (29029-1, Biotium, Fremont, CA) in 100 mM Tris, pH 8.0, 40 mM NaCl, rinsed 3x with PBS and mounted onto a custom manufactured sample holder in imaging buffer. The holder was sealed with parafilm.

### Preparation of DY634-labeled secondary anti-mouse antibody

50ul of donkey anti-mouse IgG (H+L) (1,3 mg/ml) (715-005-151, Dianova) was incubated with a 10-fold molar excess of DY634-NHS (634-01, Dyomics) in a final volume of 100ul PBS pH 7,4 overnight at RT. The labeled antibody was purified from free dye by running over an PBS equilibrated Zeba Spin desalting column (89889, Thermo Scientific) by gravity flow. Fractions containing the peak of the labeled antibody were identified by SDS-PAGE and pooled.

### Preparation of three-color synaptonemal complex samples

To prepare poly-L-lysine coated coverslips for mounting of *Caenorhabditis elegans* germline tissue, coverslips (Precision cover slips, 24 mm, PK26.1, Carl Roth GmbH), were cleaned by a 20 min wash in ethanol, rinsed in milliQ water, and plasma-cleaned (PlasmaPrep2, GaLa Instrumente GmbH). Once dry, coverslips were incubated with 0.01% (w/v) poly-L-lysine (30,000-70,000 Da, P2636-25MG, Sigma-Aldrich) for 20 min at room temperature. After incubation, coverslips were rinsed in milliQ water and left at room temperature until completely dry. Poly-L-lysine coated coverslips were stored at 4 °C until use. To obtain germline tissue, 24-h post L4 larval stage *C. elegans* worms were prepared as described previously^33^ with few modifications. After dissection, the tissue was fixed for 1 min in 2 % (v/v) FA. FA was quenched by washing three times in TBST (20 mM Tris-HCl, 150 mM NaCl, pH 7.5, 0.1% (v/v) Tween20), and by two 5 min washes in PBST (13.7 mM NaCl, 0.27 mM KCl, 1 mM Na2HPO4, 0.18 mM KH2PO4, pH 7.4, 0.1% (v/v) Tween20), followed by 1 min incubation in −20 °C methanol. Fixed samples were blocked for 45 min in 1x Roche blocking buffer PBST (11096176001, SigmaAldrich) at room temperature. Samples were then incubated with anti-HA (1:250, mouse monoclonal, 2-2.2.14, Cat. # 26183, Thermo Fisher Scientific), anti-HIM-3 (1:250, rabbit polyclonal, 53470002, Novus Biologicals), and anti-HTP-3^39^ (1:250, chicken polyclonal, gift from Prof. Dr. Abby F. Dernburg) primary antibodies diluted in Roche blocking buffer (overnight at 4 °C). Unbound primary antibodies were removed by three 10 min washes in PBST.

For 3-color SMLM imaging, primary antibodies stained samples were subsequently stained with F(ab’)2 fragments that were conjugated with fluorescent organic dyes: anti-mouse IgG (1:100, donkey polyclonal, AB_2340761, Jackson Immunoresearch) with AlexaFluor 647 (A37573, ThermoFischer Scientific), anti-rabbit IgG (1:100, donkey polyclonal, AB_2340586, Jackson Immunoresearch) with CF660C (92137, Biotium), anti-chicken IgY (1:100, donkey polyclonal, AB_2340347, Jackson Immunoresearch) with Dy-634 (634-01, Dyomics). F(ab’)2 fragments were labeled with the respective succinimidyl ester reactive dyes at a molar ratio of 1:17 for 1 hour at room temperature in PBS with 0.1 M NaHCO3 (pH 8.3). Post-labeling, F(ab’)2 fragments were separated from the residual reactive dye using a Zeba Micro Spin Desalting Column (7 K MWCO, 75 µL, 89877, ThermoFischer Scientific). Samples were incubated with these secondary antibodies in 1x Roche blocking buffer for 2 hours at room temperature. Unbound antibodies were removed with two 10 min washes in PBST. Samples were post-fixed in 2% FA (v/v) in PBS on poly-L-lysine coated coverslips, rinsed in PBS, and mounted on a custom-made holder in the imaging buffer and sealed with parafilm.

For confocal imaging, primary antibodies stained samples were stained with AlexaFluor conjugated antibodies: AlexaFluor 546 anti-mouse IgG (1:500, A10040, ThermoFischer Scientific), AlexaFluor 647 anti-rabbit IgG (1:500, donkey polyclonal, 711-605-152, Jackson Immunoresearch), AlexaFluor 488 anti-chicken IgY (1:500, donkey polyclonal, 703-545-155, Jackson Immunoresearch). Unbound antibodies were removed with two 10 min washes in PBST. The samples were mounted in ProLong Diamond Antifade (P36966, ThermoFischer Scientific) mounting medium with DAPI, and imaged on an Olympus IXplore SpinSR spinning-disk confocal microscope using a PLAPON60X (NA 1.42) oil immersion objective.

### Microscope setup

Single-objective SMLM image acquisition was performed at room temperature (RT, 24 °C) on a custom built microscope equipped with a high NA oil immersion objective (160x, 1.43-NA oil immersion, Leica, Wetzlar, Germany) described previously^40^. A commercial laser box (LightHub®, Omicron-Laserage Laserprodukte, Dudenhofen, Germany) equipped with Luxx 405, 488 and 638, Cobolt 561 lasers and an additional 640 nm booster laser (iBeam Smart, Toptica, Munich, Germany) were combined for wide field illumination. Lasers were focused onto a speckle reducer (LSR-3005-17S-VIS, Optotune, Dietikon, Switzerland) and coupled into a multi-mode fiber (M105L02S-A, Thorlabs, Newton, NJ, USA). The lasers were triggered using an FPGA (Mojo, Embedded Micro, Denver, CO, USA) allowing microsecond pulsing control of lasers. The output of the fiber was magnified by an achromatic lens and imaged into the sample plane. A laser clean-up filter (390/482/563/640 HC Quad, AHF, Tübingen, Germany) was placed in the excitation beam path to remove the fluorescence generated by the fiber. The focus of microscope was stabilized by a 785 nm infrared laser (iBeam Smart, Toptica, Munich, Germany) that was projected through the objective and reflected by the coverslip onto a quadrant photodiode, which was used as closed-loop feedback signal to the objective piezo stage (P-726 PIFOC, Physik Instrument, Karlsruhe, Germany). The fluorescence emission was filtered by a bandpass filter 676/37 (catalog no. FF01-676/37-25, Semrock) and then split into two channels (separated by ~400 nm axially) using a 50:50 beamsplitter for biplane imaging with AF647. The astigmatic 3D imaging was acquired using a cylindrical lens (f = 1,000 mm; catalog no. LJ1516L1-A, Thorlabs) to introduce astigmatism. For astigmatic multicolor imaging with DY 634, AF647, CF660C and CF680, the fluorescence of the ratiometric multi-color imaging was split by a 665 nm long pass dichroic (catalog no. ET665lp, Chroma), filtered by a 685/70 (catalog no. ET685/70 m, Chroma) bandpass filter for the transmitted light and a 676/37 (catalog no. FF01-676/37-25, Semrock) bandpass filter for the reflected light. An EMCCD camera (Evolve512D, Photometrics, Tucson, AZ, USA) was used to collect final fluorescence. The microscope is entirely controlled by Micro-Manager^41^ using htSMLM, a custom EMU plugin^42^. Typically, we acquire 100,000 – 300,000 frames with 30 ms exposure time and laser power densities of ~15 kW/cm^2^. The pulse length of the 405 nm laser is automatically adjusted to retain a constant number of localizations per frame.

4Pi-SMLM image acquisition was performed at RT based on an instrument as described previously^11^ with minor modifications. Two magnification matched silicone immersion objectives (1.35 NA, UPLSAPO, 100XS, Olympus) were used for better refractive index matching. The system was equipped with four excitation lasers: 405 nm (IBEAM-SMART-405 nm, 150 mW, Toptica), 488 nm (IBEAM-SMART-488-S-HP, 200 mW, Toptica), 560 nm (2RU-VFL-P-1500-560-B1R, MPB Communications, Pointe-Claire, Canada) and 642 nm (2RU-VFL-P-2000-642-B1R, MPB Communications). The excitation laser was filtered by a clean-up filter (390/482/563/640 HC Quad, AHF) and then reflected by a quadband dichroic (405/488/561/635, F73-867, AHF). The emission fluorescence was passed through the dichroic and then filtered by a quadband filter (432/515/595/730 HC, F67-432, AHF). The fluorescence was additionally filtered by a bandpass filter 676/37 (catalog no. FF01-676/37-25, Semrock) before collection on an sCMOS camera (ORCA-Flash 4.0v2, Hamamatsu). ~200,000 images were acquired with 25 ms exposure time. The pulse length of the 405 nm laser is automatically adjusted to retain a constant number of localizations per frame. The 4Pi-SMLM data acquisition is based on LabVIEW control software^43^.

### Statistics & reproducibility

Figures show representative data from 2 (Fig. 5a–i) or 3 (Figs. 2d, 3b, 4c, Supplementary Fig. 8) or 5 (Supplementary Fig. 7) independent experiments, or from single evaluation (Fig. 2c).

### Reporting summary

Further information on research design is available in the Nature Research Reporting Summary linked to this article.

## Supplementary information

Supplementary Information

Reporting Summary

Peer Review File

Supplementary Movie

Supplementary Software

Description of Additional Supplementary Files

## Data Availability

All raw single molecule images acquired in this work can be freely downloaded from this website: https://www.ebi.ac.uk/biostudies/studies/S-BSST839. Source data are provided with this paper.

## Source data

Source data

## Code availability

Source code for the software used in this manuscript is contained in Supplementary Software 1 and updated versions can be freely downloaded at:https://github.com/jries/SMAP/tree/develop/fit3Dcspline/GlobLochttps://github.com/Li-Lab-SUSTech/GlobLoc^44^.
