# Supplementary material for: Global fitting for high-accuracy multi-channel single-molecule localization
Source: Nat Commun. 2022 Jun 6;13:3133. doi: 10.1038/s41467-022-30719-4 (PMC9170706; doi:10.1038/s41467-022-30719-4)
Supplement: Supplementary file 1 — Supplementary Information [file 41467_2022_30719_MOESM1_ESM.pdf]

Supplementary Information:
Global fitting for high-accuracy multi-channel single-molecule localization

Yiming Li[1,2*], Wei Shi[1], Sheng Liu[2], Ivana Cavka[2,3], Yu-Le Wu[2,3], Ulf Matti[2], Decheng Wu[1], Simone Koehler[2], Jonas Ries[2,*]

[1] Department of Biomedical Engineering, Southern University of Science and Technology, Shenzhen 518055, China

[2] European Molecular Biology Laboratory, Cell Biology and Biophysics, Heidelberg 69117, Germany

[3] Collaboration for joint PhD degree between EMBL and Heidelberg University, Faculty of Biosciences

**Supplementary Figure 1**	Performance of globLoc for biplane single molecule localization
**Supplementary Figure 2**	Performance of globLoc for dual color astigmatic single molecule localization
**Supplementary Figure 3**	Performance of globLoc on 4Pi single molecule localization
**Supplementary Figure 4**	Computation speed of globLoc for Spline and Gaussian PSF model as a function of ROI size
**Supplementary Figure 5**	Photon distributions of global fit and individual fits
**Supplementary Figure 6**	Cross-talk and rejected fraction for the 4 color ratiometric imaging using DY634, AF647, CF660C and CF680
**Supplementary Figure 7**	Ratiometric dual color 3D astigmatism imaging of Nup96-SNAP-AF647 and WGA-CF680
**Supplementary Figure 8**	4 color 3D astigmatism imaging of Nup62-DY634, Nup96-AF647, ELYS-CF660C and WGA-CF680 using different reconstruction algorithms
**Supplementary Figure 9**	Settings of ThunderSTORM software used for calibrating and analyzing biplane data.
**Supplementary Table 1**	Calibration and analysis parameters that are different from the default values of ThunderSTORM software
**Supplementary Note 1**	Parameter merging for individual fits of multi-channels
**Supplementary Note 2**	Calculation of multichannel CRLB
**Supplementary Note 3**	Derivatives for experimental IAB-based 4Pi-PSF model
**Supplementary Note 4**	Method for L-M nonlinear optimization of multichannel data
**Supplementary Note 5**	GlobLoc with SMAP: a Tutorial

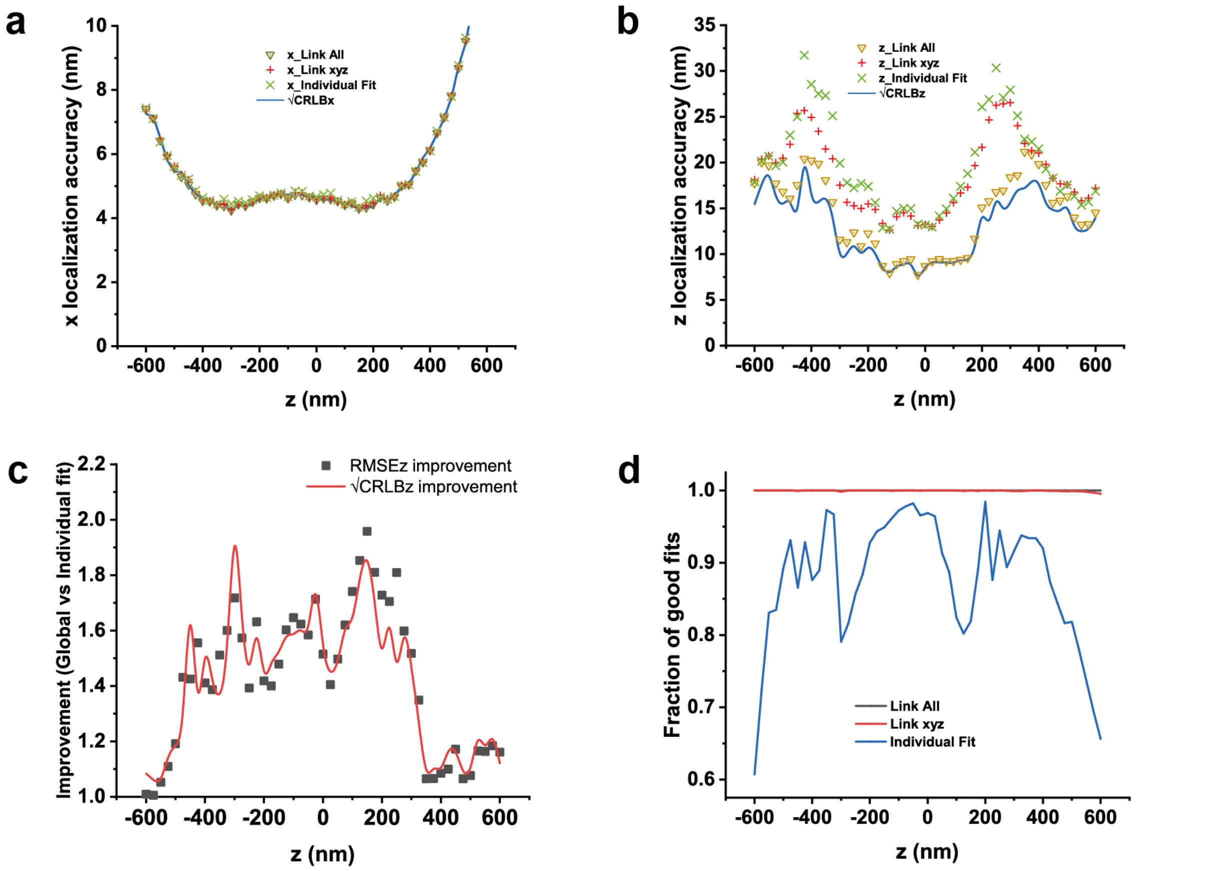

Supplementary Figure 1: Performance of globLoc for biplane single molecule localization. Biplane single molecule images were simulated using an experimental PSF from the SMLM challenge 2016 with 2,500 photons/localization and 20 background photons/pixel in each channel. **a,** *x* localization accuracy (RMSE) of dual channel single molecule data using global fit and CRLB-weighted individual fit (Supplementary Note 1). The *y* localization accuracy is similar to the *x* localization accuracy. **b,** *z* localization accuracy (RMSE) of dual channel single molecule data using global fit and CRLB-weighted individual fit. By additionally linking the photons and background parameters during fitting, the *z* localization accuracy is improved by more than 1.5 times compared to only linking *x*, *y*, and *z* positions. **c,** *z* localization accuracy improvement using global fit with all parameters linked compared to CRLB-weighted individual fit. **d,** The fraction of good fits for global fit and individual fit. If the distance between the returned *z* position and ground truth *z* position is within 8 times of the $\sqrt{\text{CRLB}}$ in the *z* direction, the fit is defined as a good fit. Only good fits were used to evaluate the localization accuracy.

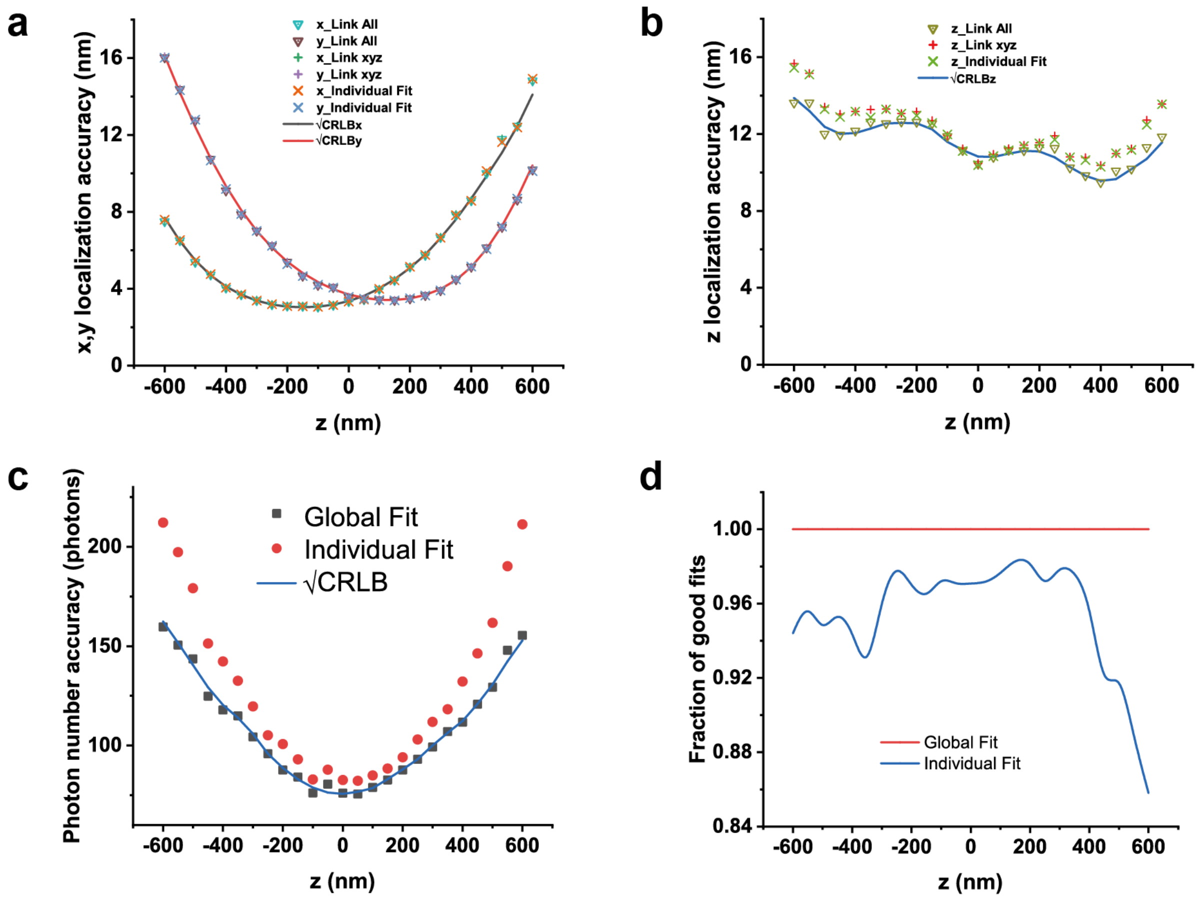

Supplementary Figure 2: Performance of globLoc for dual color astigmatic single molecule localization. Dual channel single molecule images were simulated using an experimental dual channel astigmatic PSF with 4,000 photons/localization in one channel and 1,000 photons/localization in the other channel. 20 background photons/pixel were used for both channels. **a,** *x, y* localization accuracy (RMSE) of dual channel astigmatic single molecule data using global fit and CRLB-weighted individual fits (Supplementary Note 1). **b,** *z* localization accuracy (RMSE) of dual channel astigmatic single molecule data using global fit and CRLB-weighted individual fits. **c,** Photon number accuracy (RMSE) using global fit and CRLB-weighted individual fits. **d,** The fraction of good fits for global fit and individual fits. If the distance between the returned *z* position and ground truth *z* position is within 8 times of the $\sqrt{\text{CRLB}}$ in the *z* direction, the fit defined as a good fit.

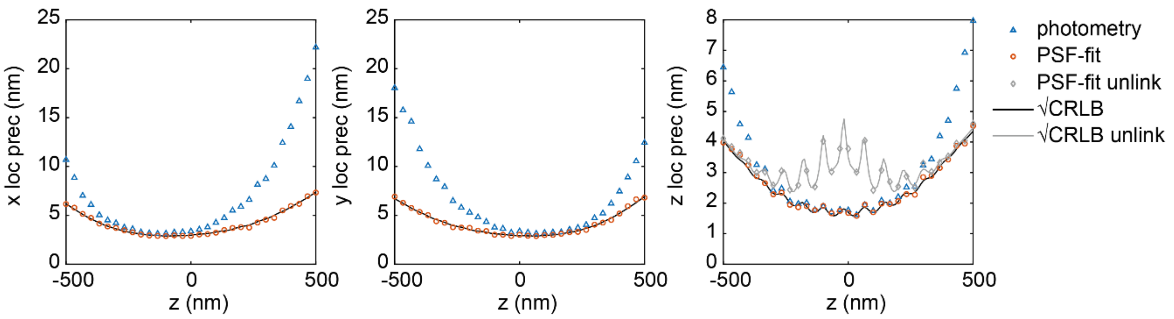

Supplementary Figure 3: Performance of globLoc on simulated 4Pi single molecule data. Comparison of localization precisions and accuracies using an experimental 4Pi-PSF model fit and the photometry-based analysis method. 1,000 4Pi single molecule images were simulated at each *z* position with four phase channels (0, π/2, π, 3π/2) with an astigmatism of 60 mλ and with *x* and *y* positions randomly distributed within -1 to 1 pixels around the center of each fitted region. For each objective, 2,000 photons/localization and 20 background photons/pixel were used. A full vectorial PSF model was used for simulations with parameters: NA 1.35. Refractive index 1.40 (immersion medium and sample) and 1.518 (cover glass). Emission wavelength 668 nm.

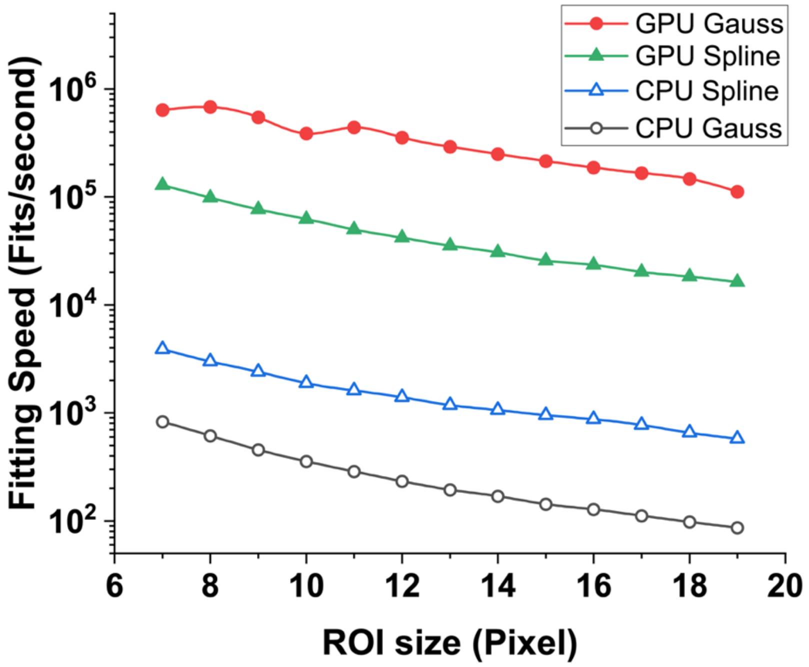

Supplementary Figure 4: Computation speed of globLoc for Spline and Gaussian PSF model as a function of ROI size. Dual channel image data with the same ROI size in each channel was simulated for speed evaluation. Fits per second were measured on an i7-8700 CPU and a RTX3090 consumer graphics card. For fitting of the spline PSF model, The GPU code is overall about 30 times faster than the CPU code running on a single thread, while it is more than 1,000 times faster for fitting of Gaussian PSF model.

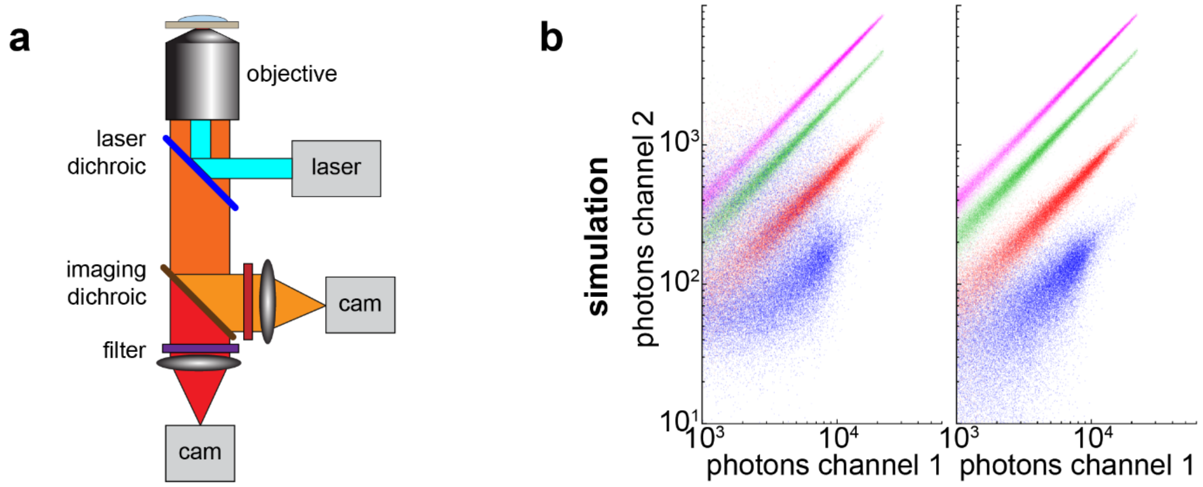

Supplementary Figure 5: Photon distributions of global fit and individual fits. a, Simplified schematic of the ratiometric multicolor SMLM imaging system. The emission fluorescence is split into two channels by a dichroic mirror. The ratio of the single molecule fluorescence between these two channels are used to determine the color information of each single molecule. **b,** Scatter plot of the fitted transmitted versus reflected photons per localization for simulated data using global fit and individual fit for DY-634, AF647, CF660C and CF680, separately. Simulations were based on experimentally derived photon ratios and photon counts (**Supplementary Fig. 6a**). The experimentally determined ratio of photons between dark and bright channels for these four dyes are 0.39, 0.21, 0.07 and 0.02.

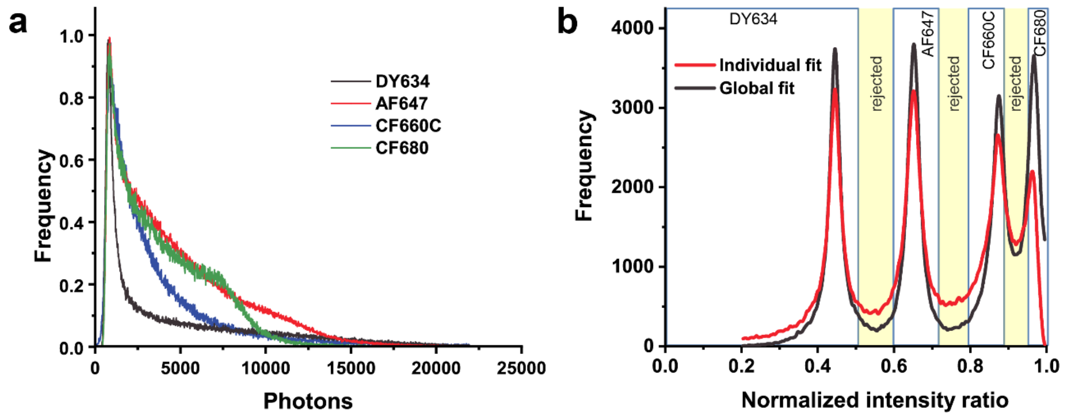

predicted dye

c (ground truth) — Global fit, Photon ratios fixed

Dyes	DY634	AF647	CF660C	CF680	Rejected
DY634	98.8	1.2	0	0	6.2
AF647	0.9	98.6	0.5	0	6.6
CF660C	0.1	1.4	93.9	4.6	30.0
CF680	0	0.1	3.2	96.7	21.5

d (ground truth) — Global fit, Link *xyz*

Dyes	DY634	AF647	CF660C	CF680	Rejected
DY634	98.5	1.5	0	0	6.5
AF647	0.9	97.8	1.0	0.1	9.6
CF660C	0.2	2.1	87.0	10.7	29.0
CF680	0	0.4	5.8	93.8	22.6

e (ground truth) — Individual fit

Dyes	DY634	AF647	CF660C	CF680	Rejected
DY634	97.6	2.3	0.1	0	7.4
AF647	6.6	91.9	1.5	0	13.6
CF660C	14.3	9.2	75.6	0.9	29.3
CF680	14.0	10.7	28.5	46.8	39.7

Supplementary Figure 6: Cross-talk and rejected fraction for the 4 color ratiometric imaging using DY634, AF647, CF660C and CF680. a, Experimental photon distributions of DY634, AF647, CF660C and CF680 in our imaging system. The photon distribution of the simulated multicolor single molecules follows the experimental photon distribution for each dye. **b**, Comparison of normalized intensity ratio between two detection channels using global fit and individual fit. The normalized intensity ratio was calculated using the following equation: $r = (I_1 - I_2)/(I_1 + I_2)$. Here, I_1 and I_2 are the fitted photons per localization for the channel 1 and channel 2, respectively. The molecules were assigned to 4 different colors based on the intensity ratio threshold indicated by the 3 boxed regions (left to right: DY634, AF647, CF660C and CF680). **c**, The cross-talk (in %) of the 4 dyes using global fit with a fixed photon ratio between channels. The *x*, *y*, *z* and photon ratio were linked. The photon ratios were determined experimentally as indicated before. Each single molecule was fitted with 4 different photon ratios separately and the solution with the maximum likelihood was chosen. Here, we filtered out molecules for which the second highest likelihood was within 0.5% of the maximum likelihood. **d** and **e** are the cross-talk of the 4 dyes analyzed by a global fit, only linking *xyz,* and by fitting the two channels separately. The molecules were filtered and assigned as described in b.

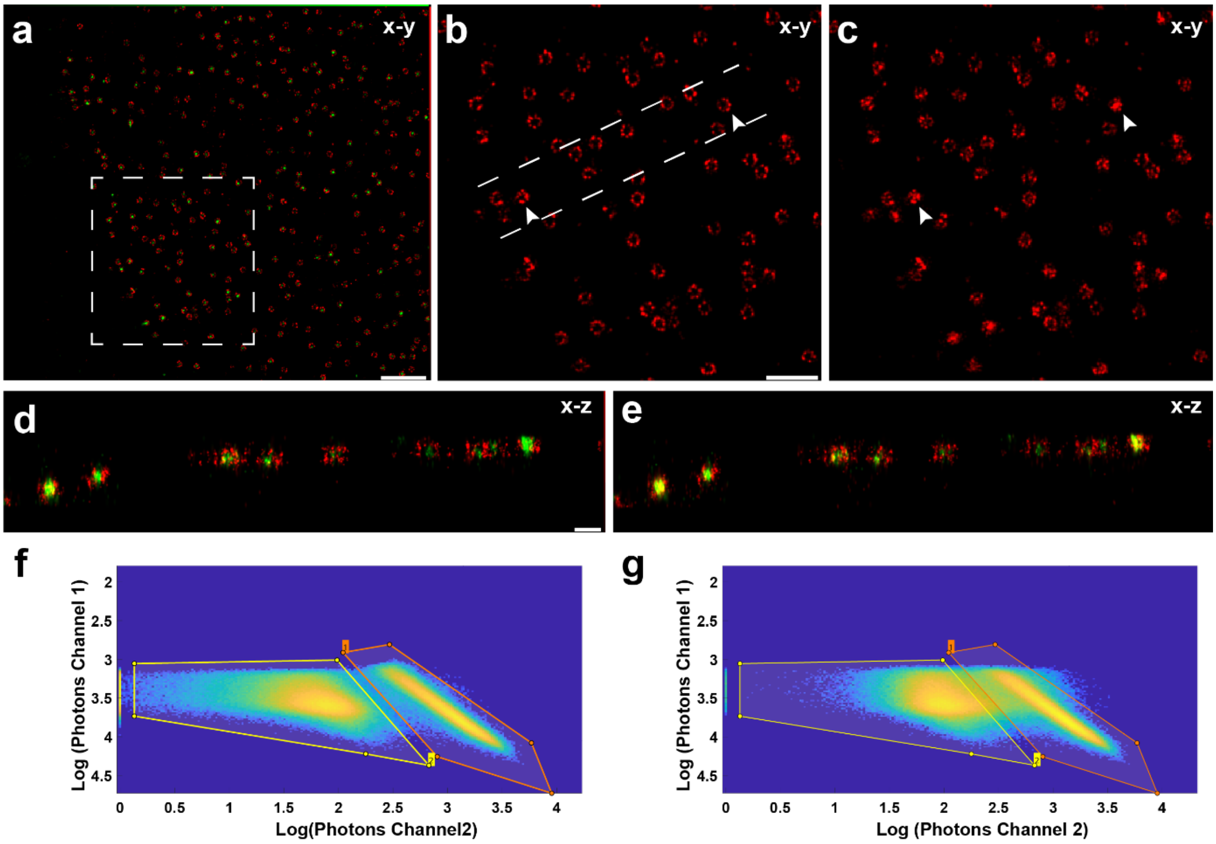

Supplementary Figure 7: Ratiometric dual color 3D astigmatic imaging of Nup96-SNAP-AF647 and WGA-CF680. a, Dual color image of Nup96 (red) and WGA (green) in a U2OS cell. Nup96 image of the boxed region in **a** reconstructed by global fit (**b**) and individual fit (**c**) separately. Arrows indicate cross-talk of the reconstructed Nup96 image contaminated by WGA. **d** and **e** are the side-view of the region bounded by dashed lines in **b** reconstructed by global fit and individual fit separately. **f** and **g** are scatter plots of the photons in bright channel versus dark channel analyzed by global fit and individual fit separately. The boxed polygons are used to assign single molecules in different colors. Representative results are shown from five experiments. Scale bars: 1 μm (**a**), 500 nm (**b**), 100 nm (**d**).

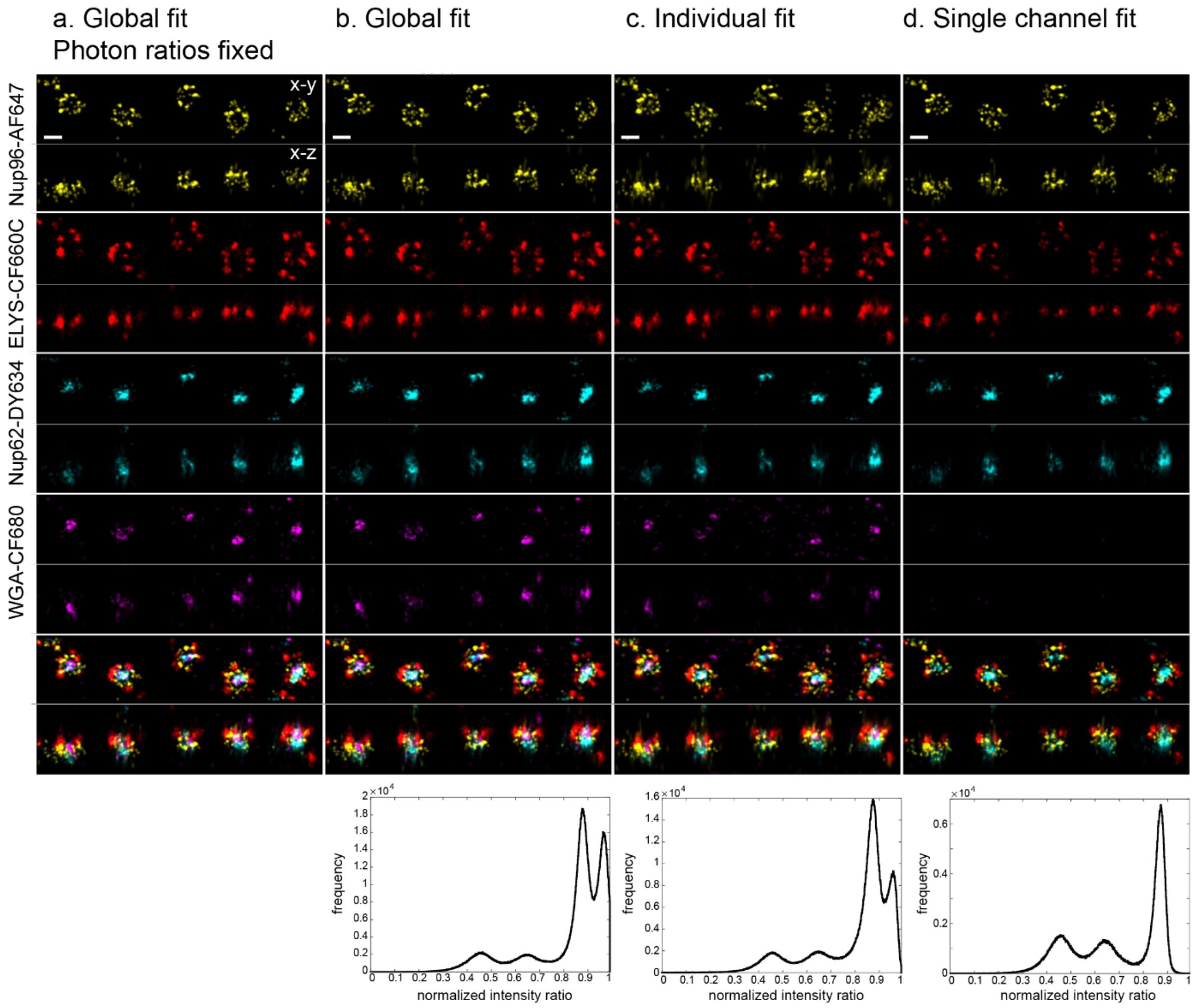

Supplementary Figure 8: 4 color 3D astigmatism imaging of Nup62-DY634, Nup96-AF647, ELYS-CF660C and WGA-CF680 using different reconstruction algorithms. a, Single molecule images are fitted with global fit with fixed photon ratios and the color was assigned based on the maximum likelihood. b, Global fit with only linking xyz and extracting the color from the ratio of fitted intensities in both channels. c, Individual fit of each channel and color assignment as in b. Here, candidate peaks from either channel are fitted also in the other channel. d, Individual fit of each channel, followed by linking corresponding localizations and color assignment as in b. As CF680 is hardly ever detected in Channel 2, it disappears from the image. Representative results are shown from 3 experiments. Scale bars 100 nm.

a: Settings of ThunderSTORM for challenge data

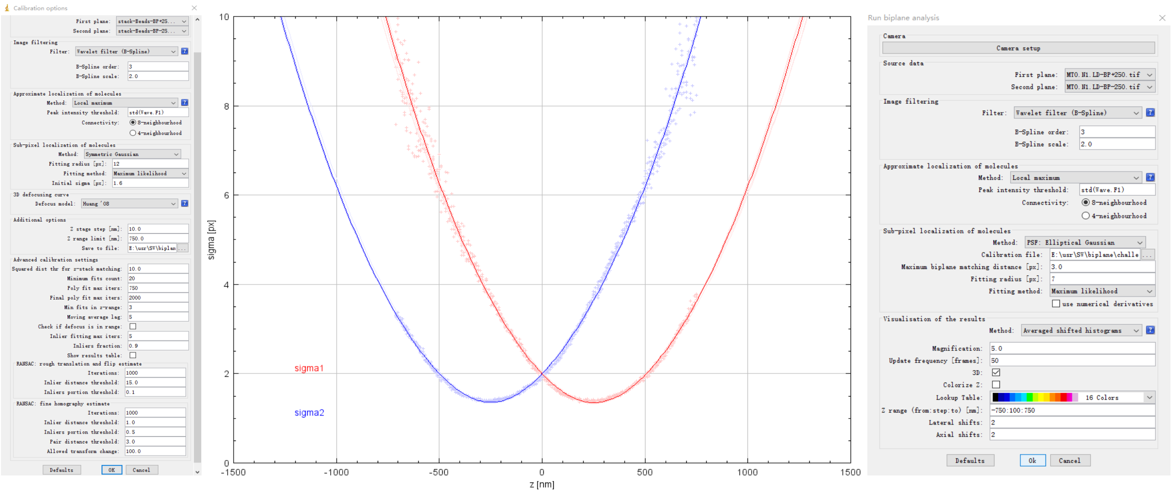

b: Settings of ThunderSTORM for experimental data

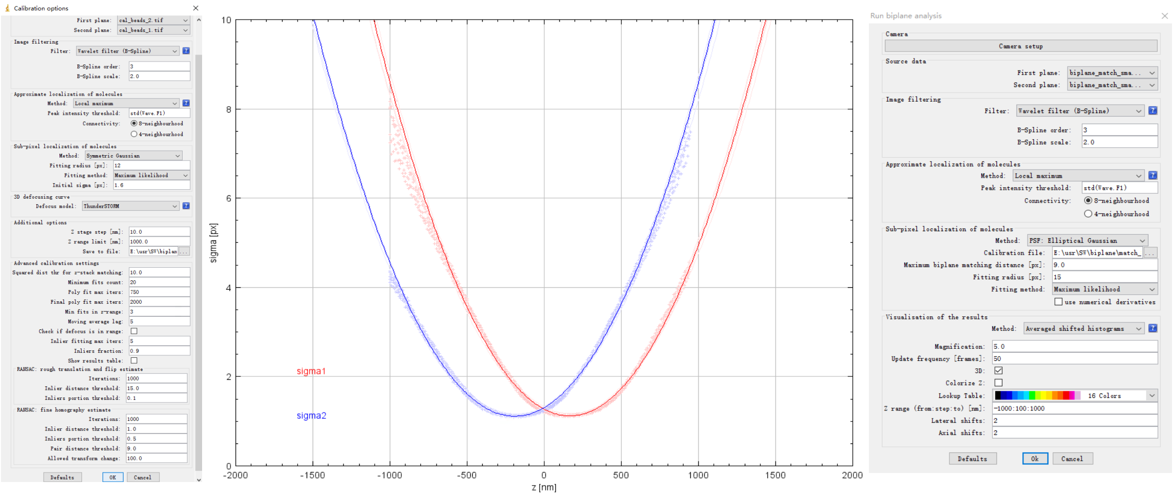

Supplementary Figure 9: Settings of ThunderSTORM software used for calibrating and analyzing biplane data. a. Left to right panel: Calibration settings of the 2016 SMLM Challenge 3D-Biplane data ranging from -750 nm to 750 nm in the axial direction, the corresponding calibration curve and analysis settings of the challenge training biplane datasets (MT0.N1.LD_BP). The reconstructed SMLM image is shown in **Figure 2c4**. **b**. Left to right panel: Calibration settings of an experimental *z*-stack beads ranging from -1 µm to 1 µm in the axial direction, the corresponding calibration curve and analysis settings of the experimental biplane datasets (Nup96-AF647). The reconstructed SMLM image is shown in **Figure 2d4**.

Supplementary Table 1

	parameter	challenge data	experimental data
Calibration parameters	Fitting radius[px]	12	12
	Defocus model	Huang’08	ThunderSTORM
	Z range limit[nm]	750	1000
	Pair distance threshold	3	9
Biplane analysis parameters	Maximum biplane matching distance[px]	3	9
	Fit radius[px]	7	15
	Z rang(from:step:to)[nm](3D)	-750:100:750	-1000:100:1000

Calibration and analysis parameters that are different from the default values of ThunderSTORM software (ThunderSTORM version: dev-2016-09-10-b1, github.com/zitmen/thunderstorm/releases/tag/dev-2016-09-10-b1).

Supplementary Note 1: Parameter Merging for individual fits of multi-channels

To merge parameters returned from individual fits of different channels, weighted arithmetic mean of parameters from all channels was used. Here, we used the reciprocal of the estimated CRLB as the weights for each parameter:

$$\theta_m = (\sum_i \theta_{mi}/CRLB_{\theta_{m,i}})/(\sum_i 1/CRLB_{\theta_{mi}})). \quad \text{(s1)}$$

Here, θ_{mi} is the set of parameters being estimated in the i_{th} channel and $CRLB_{\theta_{mi}}$ is the corresponding CRLB. The significance of this choice is that this weighted mean is the maximum likelihood estimator of parameters of different channels under the assumption that they are independent and normally distributed with the same mean. Therefore, this combination could return the optimized localization precision. As show in Supplementary Fig. 1a and Supplementary Fig. 2a, the localization accuracy of the global fit with linking *xyz* is similar to that of the CRLB-weighted individual fits. However, the global fit could substantially improve the localization accuracy in *z* by additionally linking photon parameters.

Supplementary Note 2: Calculation of multichannel CRLB

To quantify the localization precision of globLoc fitter, we compared it with the CRLB which is the limiting lower bound of the variance for any unbiased estimator. The general definition of CRLB is evaluated as the diagonal element of the inverse of the Fisher information matrix:

$$var(\hat{\theta}) \geq I(\theta)^{-1}, \quad \text{(s2)}$$

where $var(\hat{\theta})$ is the variance of an estimator and $I(\theta)$ is the Fisher information matrix. Depending on how the parameters are linked during fit, the Fisher information matrix is defined as

$$I_{m,n} = E\left[\sum_i \sum_k \frac{\partial \chi^2_{mle}}{\partial \theta_{mi}} \frac{\partial \chi^2_{mle}}{\partial \theta_{ni}}\right] = \begin{cases} \sum_i \sum_k S_{mi} \frac{\partial u_{ki}}{\partial \theta_{mp}} S_{ni} \frac{\partial u_{ki}}{\partial \theta_{np}} \frac{1}{u_{ki}}, & \theta_{mi} \in \boldsymbol{\theta_p}, \theta_{ni} \in \boldsymbol{\theta_p} \\ \sum_k S_{mi} \frac{\partial u_{ki}}{\partial \theta_{mp}} \frac{\partial u_{ki}}{\partial \theta_{ni}} \frac{1}{u_{ki}}, & \theta_{mi} \in \boldsymbol{\theta_p}, \theta_{ni} \in \boldsymbol{\theta_{qi}} \\ \sum_k \frac{\partial u_{ki}}{\partial \theta_{mi}} \frac{\partial u_{ki}}{\partial \theta_{ni}} \frac{1}{u_{ki}}, & \theta_{mi} \in \boldsymbol{\theta_{qi}}, \theta_{ni} \in \boldsymbol{\theta_{qi}}, m = n \\ 0, & \theta_{mi} \in \boldsymbol{\theta_{qi}}, \theta_{ni} \in \boldsymbol{\theta_{qi}}, m \neq n \end{cases}.$$

(s3)

Here, χ^2_{mle} is defined as Equation (1) in Methods, μ_{ki} is the expected photon number in the *k*th pixel of the *i*th channel. $\boldsymbol{\theta_p}$ is the set of global parameter and $\boldsymbol{\theta_{qi}}$ is the set of local parameters of *i*th channel.

Supplementary Note 3: Derivatives for IAB-based 4Pi-PSF model

The IAB-based 4Pi-PSF model is written as in ref. [1]:

$$P(x, y, z, \varphi) = \left(E_1 + E_2 e^{-i\varphi}\right)\left(E_1 + E_2 e^{-i\varphi}\right)^*$$
$$= |E_1|^2 + |E_2|^2 + E_1 E_2^{*} e^{i\varphi} + E_1^{*} E_2 e^{-i\varphi}$$
$$= I + A\cos(\varphi) + B\sin(\varphi). \tag{s4}$$

Here, φ is the interference phase. $I(x, y, z)$, $A(x, y, z)$ and $B(x, y, z)$ are phase independent and slowly varying real functions of x, y, z. In order to construct the Hessian and Jacobian matrix, the following partial derivatives of x, y, z, phase, photons and background are used:

$$\frac{\partial\mu_{ki}}{\partial\theta_x} = \theta_N\left(\frac{\partial \mathrm{I}(x-\theta_x, y-\theta_y, \theta_z)}{\partial\theta_x} + \frac{\partial \mathrm{A}(x-\theta_x, y-\theta_y, \theta_z)}{\partial\theta_x}\cos\left(\theta_\varphi + \phi_i\right) + \frac{\partial \mathrm{B}(x-\theta_x, y-\theta_y, \theta_z)}{\partial\theta_x}\sin\left(\theta_\varphi + \phi_i\right)\right), \tag{s5}$$

$$\frac{\partial\mu_{ki}}{\partial\theta_y} = \theta_N\left(\frac{\partial \mathrm{I}(x-\theta_x, y-\theta_y, \theta_z)}{\partial\theta_y} + \frac{\partial \mathrm{A}(x-\theta_x, y-\theta_y, \theta_z)}{\partial\theta_y}\cos\left(\theta_\varphi + \phi_i\right) + \frac{\partial \mathrm{B}(x-\theta_x, y-\theta_y, \theta_z)}{\partial\theta_y}\sin\left(\theta_\varphi + \phi_i\right)\right), \tag{s6}$$

$$\frac{\partial\mu_{ki}}{\partial\theta_z} = \theta_N\left(\frac{\partial \mathrm{I}(x-\theta_x, y-\theta_y, \theta_z)}{\partial\theta_z} + \frac{\partial \mathrm{A}(x-\theta_x, y-\theta_y, \theta_z)}{\partial\theta_z}\cos\left(\theta_\varphi + \phi_i\right) + \frac{\partial \mathrm{B}(x-\theta_x, y-\theta_y, \theta_z)}{\partial\theta_z}\sin\left(\theta_\varphi + \phi_i\right)\right), \tag{s7}$$

$$\frac{\partial\mu_{ki}}{\partial\theta_\varphi} = \theta_N\left(-\mathrm{A}\left(x-\theta_x, y-\theta_y, \theta_z\right)\sin\left(\theta_\varphi + \phi_i\right) + \mathrm{B}\left(x-\theta_x, y-\theta_y, \theta_z\right)\cos\left(\theta_\varphi + \phi_i\right)\right), \tag{s8}$$

$$\frac{\partial\mu_{ki}}{\partial\theta_N} = \mathrm{I}\left(x-\theta_x, y-\theta_y, \theta_z\right) + \mathrm{A}\left(x-\theta_x, y-\theta_y, \theta_z\right)\cos\left(\theta_\varphi + \phi_i\right) + \mathrm{B}\left(x-\theta_x, y-\theta_y, \theta_z\right)\sin\left(\theta_\varphi + \phi_i\right), \tag{s9}$$

$$\frac{\partial\mu_{ki}}{\partial\theta_{bi}} = 1 \tag{s10}$$

Here, the cubic splines are used to interpolate the 3D matrices I, A and B to calculate the partial derivative along x, y and z, separately[2,3]. The L-M iterative process is considered to be converged when the ratio of the relative change of χ^2_{mle} is less than 10^{-6} compared to the last iteration.

Supplementary Note 4: Method for L-M nonlinear optimization of multichannel data

For maximum likelihood estimation, the cost function χ^2_{mle} is defined as Equation (1) in Methods. During the L-M optimization process, Hessian ($H_{m,n}$) and Jacobian (J_m) matrix are defined as Equation (4) and (5) in the Methods. The detailed optimization algorithm used in this work is described below:

1. Calculate χ^2_{mle}(old) with a user defined starting parameters θ_m.
2. Initialize λ as 0.1. λ is a damping factor that controls whether the L-M fit should behave more as a gradient descent fit method (lambda<<1) or an expansion fit method (lambda>>1).
3. Calculate the updates of each parameter $\Delta\theta_m$ by solving the linear equations:

1. $(\boldsymbol{H} + \lambda \boldsymbol{I})\Delta\boldsymbol{\theta} = \boldsymbol{J}$.

4. Derive new trial-fit-parameters $\theta_m(new)$ as follows:

2. $\theta_m(new) = \theta_m(old) + \frac{\Delta\theta_m}{1+abs\left(\frac{\Delta\theta_m}{Max_{\theta_m}}\right)}$.

3. Here, Max_{θ_m} is a clamping factor which controls the maximum change of parameter θ_m during each iteration. If the sign of $\Delta\theta_m$ has changed since last update, Max_{θ_m} is multiplied by 0.5 to suppress oscillations during optimization and damp excessively large corrections[4].

5. Determine χ^2_{mle}(new) with the new parameter using Equation (1) in the Methods.
6. If $\chi^2_{mle}(new) < \chi^2_{mle}(old)$, substitute λ with λ/10, and $\theta_m(old)$ with $\theta_m(new)$ and continue with step 3.
7. If $\chi^2_{mle}(new) \geq 1.5 * \chi^2_{mle}(old)$, substitute λ with 10λ, keep $\theta_m(old)$ unchanged, and continue with step 3.
8. If $\chi^2_{mle}(old) \leq \chi^2_{mle}(new) < 1.5 * \chi^2_{mle}(old)$, keep λ unchanged, substitute $\theta_m(old)$ with $\theta_m(new)$, and continue with step 3.

This iterative calculation may be stopped when one of the following conditions is met:

a. The ratio of the relative change of $\chi^2_{mle}(new)$ and $\chi^2_{mle}(old)$ decreases to less than a specified value (10^{-6} in this work).
b. The iteration time of the calculation loops exceeds a maximum number.

Supplementary Note 5: GlobLoc with SMAP, a tutorial

Getting started 15
Example: 4 color 3D Nuclear pore complex 15
Bead calibration 16
Global fitting with SMAP 16
Global fitting with fixed photon ratios 16
Post-processing and Color assignment 17
Rendering 17
Filtering 17
Drift correction 17
Color assignment 17
3D visualization 18
Example: bi-plane SMLM of the nuclear pore complex 18
Bead calibration 18
Fit bi-plane SMLM data with SMAP 19
Post-processing 20
Filtering 20
Drift correction 20
Rendering 20
Using globLoc in own software 20

Getting started

Here, we show you how to use global fitting in SMAP. You find a link to the Matlab and compiled versions to SMAP as well as the documentation and example data at: www.rieslab.de/#software.

Please follow the SMAP *User Guide* for installation and detailed description of general workflows. We also recommend you to follow the *Getting Started Guide* with examples. The tutorial was tested under Matlab 2019a with CUDA 11.3 in Win10 system. A typical install time is less than 10 minutes.

Installing CUDA toolkit 11.3

Download CUDA 11.3, Windows 10, network installer from:

4. https://developer.nvidia.com/cuda-11.3.0-download-archive

and Install CUDA using the default installation option, which is the "Express" installation.

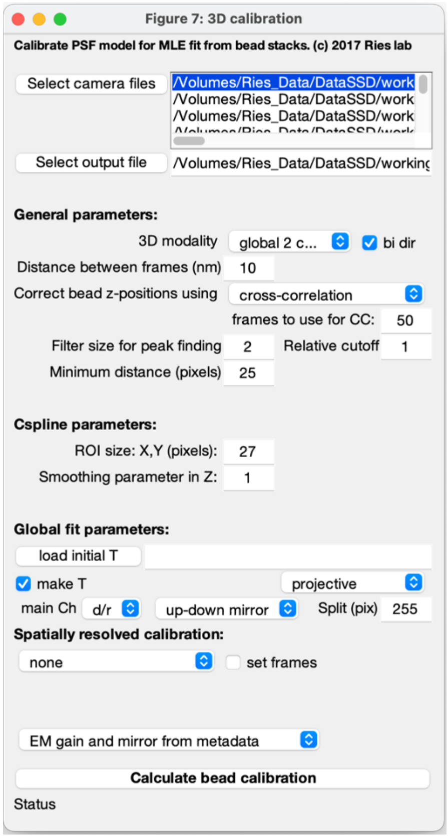

Figure 1: Settings for the plugin: calibrate3DsplinePSF.

Example: 4 color 3D Nuclear pore complex

The example files for globLoc you can find at: https://www.ebi.ac.uk/biostudies/studies/S-BSST839. To analyze the 4-color 3D nuclear pore complex (NPC) data,

please download and extract the NPC4C.zip. Please note that this is only a subset of the frames used to for Figure 2 in the manuscript.

Bead calibration

Compare section 5.4 in the *User Guide*.

1. Start SMAP
2. Select the **calibrate3DsplinePSF** plugin from the **Menu/Analyze/sr3d** or in the **Analyze/sr3D** tab and press Run.
3. Load bead images with select camera files:
   a. You can add individual bead stacks individually with add or add an entire directory containing many bead stacks with add dir.
   b. Set the following parameters according to Figure 1.
   c. Calculate bead calibration.

Global fitting with SMAP

For general settings (single-channel fitting) that are also relevant for globLoc see the *User Guide* section 5.

4. In the localize tab in the bottom press Change to load the workflow: `fit_global_dualchannel.txt`
5. load images: select any of the individual image tiff files.
6. Check if metadata has been set by clicking on set Cam Parameters. Importantly, the coordinates of the ROI need to be: [100, 0, 287, 512].
7. In the **Peak Finder** tab load the transformation with load T and select the bead calibration file that you created before, i.e. `beads_3dcal.mat`.
8. In the **Fitter** tab load 3D cal and select the same file again.
9. Set the parameters according to Figure 2.
10. You can select in the table on the right had side which parameters to link during the fit (here: x,y,z).
11. Check if fitting works with Preview. You can adjust the cutoff (**dynamic factor**) in the peak finder to fit more or less candidates.
12. Press Localize to start the fitting. Depending on the computer and if the GPU or CPU are used, this can take between a few minutes and an hour.

Global fitting with fixed photon ratios

To fit ratiometric mulit-color data with fixed photon ratios, follow points 4-12 from above, but check the Multi color: fix ratio to: checkbox and insert the intensity ratios of the different dyes as a list (Figure 3). In addition, make sure to link *N* in the list. After fitting, the color is saved as the field *color*. *LLsecond* denotes the ratio of the second highest log-likelihood to the

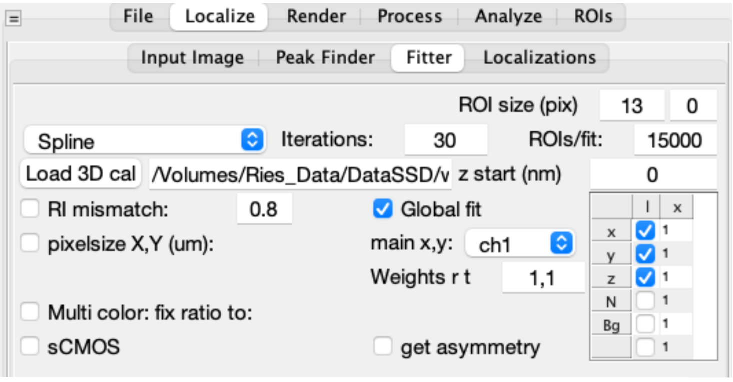

Figure 2: Parameters for the fitting workflow.

hightest. If this value is close to 1, the color assignment is not certain. You can use this field for filtering to choose between a high precision and a low rejection.

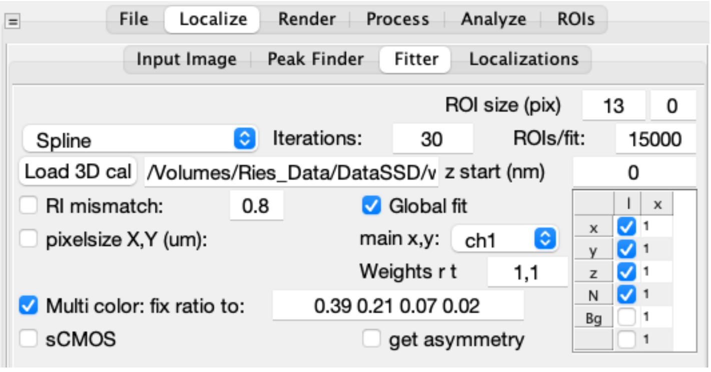

Figure 3: Parameters for fitting with fixed photon ratios

Post-processing and Color assignment

Rendering

When the fitting is done you can render the super-resolution image. To this end, press Render in the **Render** tab. Please consult the *User Guide* section 7 for detailed instructions and familiarize yourself with the rendering.

Filtering

Use the versatile filtering options of SMAP (*User Guide* section 6.2) to only display localizations of sufficient quality (we recommend filtering by the lateral localization precision to only display bright localization events, and by the relative log-likelihood (LLrel) to reject erroneous localizations from fluorophores activated too close to each other.

Drift correction

You can perform a 3D drift correction based on redundant cross-correlation as explained in the *User Guide* section 6.3.

Color assignment

Here we discuss, how to assign to each localization its color (compare *User Guide* section 9.2.2). This is not necessary when you fitted with fixed intensity ratios:

Open the plugin **Process/Assign2C/Intensity2ManyChannels.** This plugin assigns color values to the localizations based on two fields of the localization data, here the photon numbers fitted in channel 1 (phot1) and channel 2 (phot2), respectively. These values are assigned to the channel field of the localizations.

13. Select the two fields that encode the intensity in both channels, here phot1 and phot2. From this selection an image is generated in which the x-axis corresponds to the first intensity value and the y-axis corresponds to the second intensity value (logarithmic scaling). By checking log scale you can also use a logarithmic scaling for the contrast.
14. Press ROI 1 to draw a polygon ROI around the area the image that encloses all localizations corresponding to Color 1 (Figure 4).
15. Repeat the same for Color 2 and additional Colors (for Color 4 and onwards put the color number in the respective field).
16. With Show ROIs you can display all ROIs. You can always adjust all ROIs.

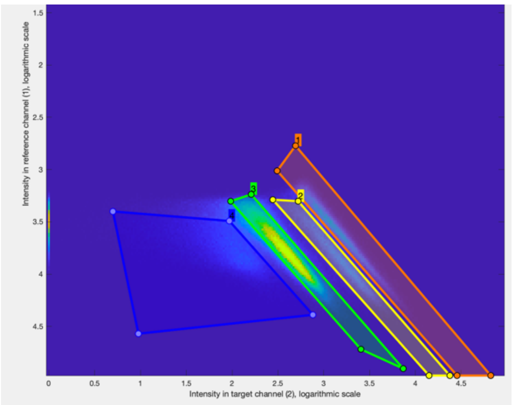
Figure 4: Dual color assignment. ROIs used to assign four colors to the intensity ratios.

17. With Delete ROIs you can delete all ROIs and start over.
18. With load and save you can load and save all defined ROIs for later use.
19. Press Run to assign a Color number to all localizations based on the defined ROIs.
    a. If use grouped is selected, this assignment is based on grouped localizations.
    b. Localizations outside of any ROI are assigned the channel value 0. This is also the channel value for all data before channel assignment.
20. In the **Render** tab please create 3 additional layers with the + tab.
    a. In Layer 1, put in 1 for the Color in the Ch field. For Layers 2-4 put the color values 2-4.
    b. In each layer, choose a different lookup table (LUT).
21. Now you should be able to render a four-color image.
22. In the 'layers' panel on the top-right side of the GUI you can select which layers to display, and also if to show the layers separately (split) and if to show in addition the composite image (comp).

3D visualization

For 3D visualization with the SMAP 3D viewer please follow the *User Guide* section 7.6.

Example: bi-plane SMLM of the nuclear pore complex

To analyze the bi-plane 3D nuclear pore complex (NPC) data, please download and extract the NPC_BP.zip from https://www.ebi.ac.uk/biostudies/studies/S-BSST839.

Bead calibration

1. Start SMAP
2. Select the ***calibrate3DsplinePSF*** plugin from the ***Menu/Analyze/sr3d*** or in the ***Analyze/sr3D*** tab and press Run.
3. Select camera files to load all the bead stacks. A new window opens. Here press add dir and select all directories in the bead directory. Press Done
4. Set the parameters according to Figure 5.
    a. As we have bi-plane data in two parts of the camera we choose the 3D modality: global 2 channel.
    b. For nicer visualization of the validation of the model you can select ☐ bi dir to activate bi-directional fitting.
    c. Set the distance between slices in the stack (in nanometers).
    d. You can adjust the other parameters according to the *User guide* section 5.4.1.
    e. An initial transformation is calculated from the bead stacks. You can choose the main channel (in this case the bottom channel) and specify that the upper and lower channels are mirrored horizontally with respect to each other.

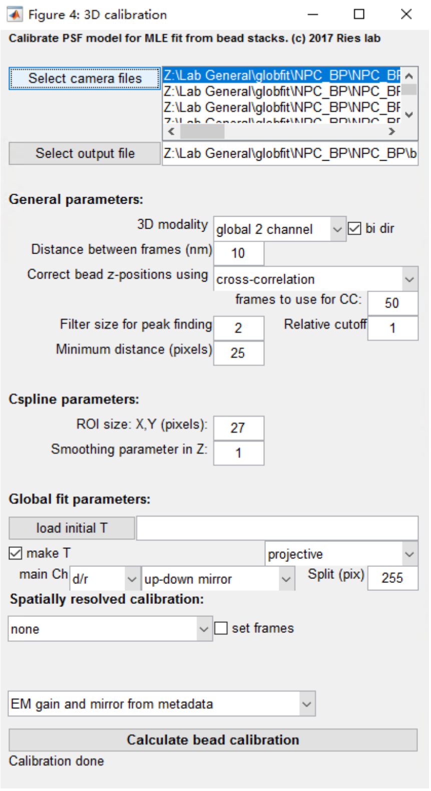

Figure 5: Settings for the plugin: calibrate3DsplinePSF.

f. We let SMAP determine the EM gain and mirror from the metadata. Otherwise, set here if beads were acquired with the conventional or EM mode.

5. Calculate bead calibration. This step might take 5-10 minutes, you can see the progress in the status bar of the plugin. You can compare your saved files with beads_BP_3dcal.fig. Specifically, compare the tabs of the output figure with those in Figure 6.

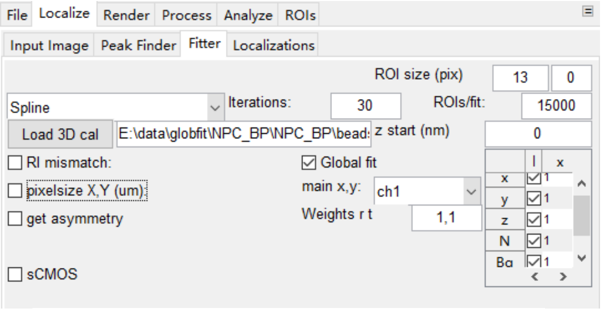

Figure 7: Parameters for the fitting workflow

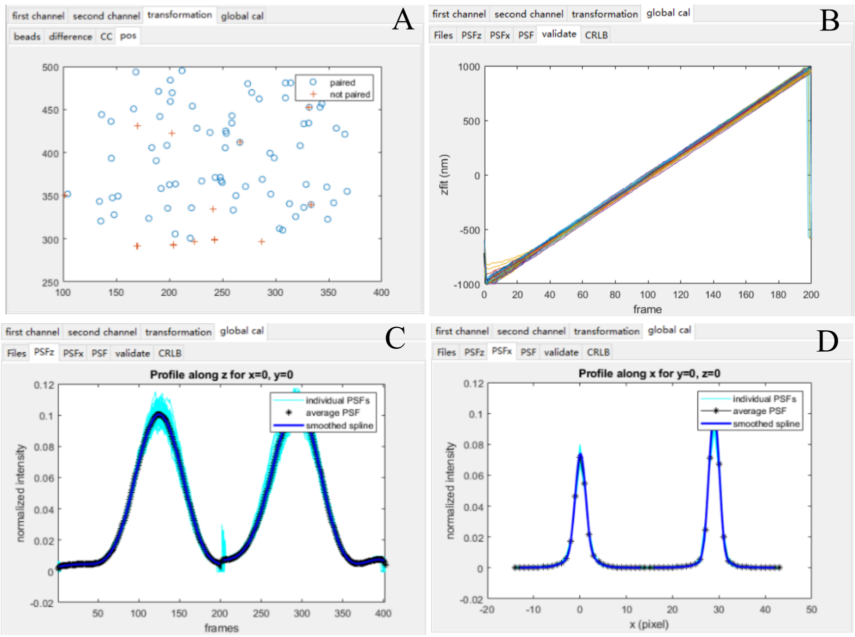

Figure 6: Output of the GUI calibration. A) to check if beads across the whole field of view were paired. B) to check if the model could properly fit the individual beads. C) Cross-section along z-axis through model to check if the model represents the individual beads. D) as C. but along x-axis.

Fit bi-plane SMLM data with SMAP

For general settings (single-channel fitting) that are also relevant for globLoc see the User Guide section 5.

6. Check in the SMAP GUI at the bottom of the Localize tab that the fitting workflow fit_global_dualchannel is selected. Otherwise load that workflow by pressing Change.
7. In the main SMAP GUI select the Localize/Input Image tab.
8. Load images and select one of the files in the bi_plane data directory .The loader might display an error message that it cannot load the images. Ignore this for the moment and try the next steps. This error message comes from the micro-manager code, thus it cannot easily be fixed in SMAP.
9. Check if metadata has been set by clicking on set Cam Parameters. Importantly, the coordinates of the ROI need to be: [2, 0, 512, 512].
10. In the Peak Finder tab load the transformation with load T and select the bead calibration file that you created before, i.e. beads_BP_3dcal.mat.

11. In the Fitter tab load 3D cal and select the same file again.
12. You can select in the table on the right had side (Figure 7) which parameters to link during the fit (here: x,y,z,N,Bg).
13. You can adjust the parameters in the Peak Finder tab according to the User guide section 5.3.
14. Check if fitting works with Preview. You can adjust the cutoff (dynamic factor) in the peak finder to fit more or less candidates.
15. Press Localize to start the fitting. Depending on the computer and if the GPU or CPU are used, this can take between a few minutes and an hour.

Post-processing

Filtering

Use the versatile filtering options of SMAP (User Guide section 6.2) to only display localizations of sufficient quality (we recommend filtering by the lateral localization precision to only display bright localization events, and by the relative log-likelihood (LLrel) to reject erroneous localizations from fluorophores activated too close to each other).

Drift correction

You can perform a 3D drift correction based on redundant cross-correlation as explained in the User Guide section 6.3.

Rendering

You can render the super-resolution image in the Render tab (Figure 8). Please consult the User Guide section 7 for detailed instructions and familiarize yourself with the rendering.

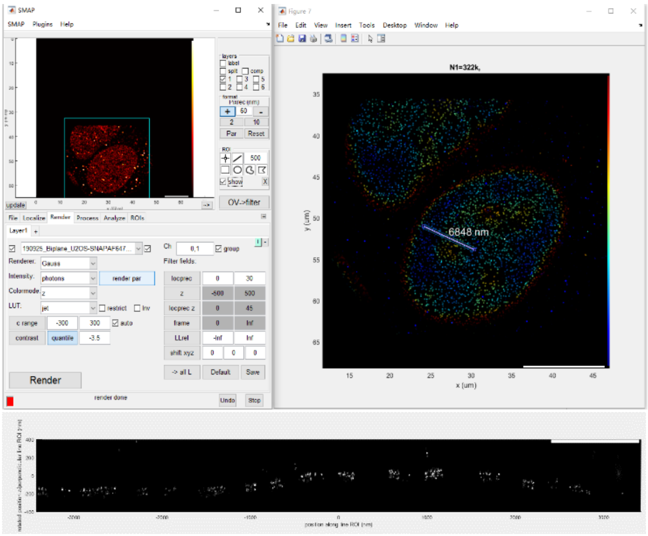

Figure 8: GUI panels to render render the super-resolution image and side-view rendering of the localizations in the line ROI.

Using globLoc in own software

GlobLoc is designed as an independent module that can be integrated with custom software. We implemented the CUDA C/C++ source code as dynamic-link libraries which can be directly called from MATLAB and Python environments. For both MATLAB and Python, we provide 4 example codes for biplane, ratiometric, Gaussian and 4Pi single molecule localization as a template for using globLoc in own software. The example code can be found at:

https://github.com/Li-Lab-SUSTech/GlobLoc

and https://github.com/jries/SMAP/tree/develop/fit3Dcspline/GlobLoc.

References

1. Li, Y. *et al.* Accurate 4Pi single-molecule localization using an experimental PSF model. *Opt. Lett.* **45**, 3765-3768 (2020).
2. Li, Y. *et al.* Real-time 3D single-molecule localization using experimental point spread functions. *Nat. Methods* **15**, 367–369 (2018).
3. Babcock, H. P. & Zhuang, X. Analyzing Single Molecule Localization Microscopy Data Using Cubic Splines. *Sci. Rep.* **7**, 552 (2017).
4. Tobergte, D. R. & Curtis, S. Daophot: a Computer Program for Crowded-Field Stellar Photometry (Psf Photometry). *J. Chem. Inf. Model.* **53**, 1689–1699 (2013).
