# Supplementary material for: Global fitting for high-accuracy multi-channel single-molecule localization
Source: Nat Commun. 2022 Jun 6;13:3133. doi: 10.1038/s41467-022-30719-4 (PMC9170706; doi:10.1038/s41467-022-30719-4)
Supplement: Supplementary file 2 — Reporting Summary [file 41467_2022_30719_MOESM2_ESM.pdf]

nature research

Corresponding author(s): Yiming Li and Jonas Ries

Last updated by author(s): Apr 20, 2022

Reporting Summary

Nature Research wishes to improve the reproducibility of the work that we publish. This form provides structure for consistency and transparency in reporting. For further information on Nature Research policies, see our Editorial Policies and the Editorial Policy Checklist.

Statistics

For all statistical analyses, confirm that the following items are present in the figure legend, table legend, main text, or Methods section.

n/a	Confirmed	
☐	☒	The exact sample size (*n*) for each experimental group/condition, given as a discrete number and unit of measurement
☐	☒	A statement on whether measurements were taken from distinct samples or whether the same sample was measured repeatedly
☒	☐	The statistical test(s) used AND whether they are one- or two-sided<br>*Only common tests should be described solely by name; describe more complex techniques in the Methods section.*
☒	☐	A description of all covariates tested
☒	☐	A description of any assumptions or corrections, such as tests of normality and adjustment for multiple comparisons
☐	☒	A full description of the statistical parameters including central tendency (e.g. means) or other basic estimates (e.g. regression coefficient) AND variation (e.g. standard deviation) or associated estimates of uncertainty (e.g. confidence intervals)
☒	☐	For null hypothesis testing, the test statistic (e.g. *F*, *t*, *r*) with confidence intervals, effect sizes, degrees of freedom and *P* value noted<br>*Give P values as exact values whenever suitable.*
☒	☐	For Bayesian analysis, information on the choice of priors and Markov chain Monte Carlo settings
☒	☐	For hierarchical and complex designs, identification of the appropriate level for tests and full reporting of outcomes
☒	☐	Estimates of effect sizes (e.g. Cohen's *d*, Pearson's *r*), indicating how they were calculated

Our web collection on statistics for biologists contains articles on many of the points above.

Software and code

Policy information about availability of computer code

Data collection	Micro-Manager (1.4 and 2.0),available at http://micro-manager.org<br>htSMLM custom software available at https://github.com/jdeschamps/htSMLM,<br>National Instruments (NI) LabVIEW 2016 64-bit ,custom software available at https://github.com/Gurdon-Super-Res-Lab/Microscope-Control
Data analysis	Matlab R2019a ,<br>Windows Visual Studio 2019 with CUDA 11.3 ,<br>Python 3.8 ,<br>Fiji ,ThunderSTORM (version dev-2016-09-10-b1) available at https://github.com/zitmen/thunderstorm/releases/tag/dev-2016-09-10-b1,<br>SMAP custom software available at https://github.com/jries/SMAP and https://github.com/Li-Lab-SUSTech/GlobLoc

For manuscripts utilizing custom algorithms or software that are central to the research but not yet described in published literature, software must be made available to editors and reviewers. We strongly encourage code deposition in a community repository (e.g. GitHub). See the Nature Research guidelines for submitting code & software for further information.

Data

Policy information about availability of data

All manuscripts must include a data availability statement. This statement should provide the following information, where applicable:

- Accession codes, unique identifiers, or web links for publicly available datasets
- A list of figures that have associated raw data
- A description of any restrictions on data availability

The experimental biplane and 4 color 3D astigmatic datasets can be freely downloaded from this website: https://www.embl.de/download/ries/globLoc/. All other data are available upon reasonable request from the corresponding authors Yiming Li and Jonas Ries upon request.

Field-specific reporting

Please select the one below that is the best fit for your research. If you are not sure, read the appropriate sections before making your selection.

- [x] Life sciences
- [ ] Behavioural & social sciences
- [ ] Ecological, evolutionary & environmental sciences

For a reference copy of the document with all sections, see nature.com/documents/nr-reporting-summary-flat.pdf

Life sciences study design

All studies must disclose on these points even when the disclosure is negative.

Sample size	No sample-size calculation was performed. The manuscript reports the demonstration of an imaging method, but draws no biological conclusions, and does not examine or compare different biological conditions. This is not a life science study with comparative analyses of a certain sample size. We always chose a sample size that validates reproducibility of our technique.
Data exclusions	No data was excluded from the analysis.
Replication	All attempts at replication were successful. All experiments were repeated three or more times with similar results.
Randomization	No randomization was performed. Randomization was not necessary, because his is not a life science study with comparative analyses of biological situations.
Blinding	No blinding was performed. There is no comparison of different biological situations performed in this work.

Reporting for specific materials, systems and methods

We require information from authors about some types of materials, experimental systems and methods used in many studies. Here, indicate whether each material, system or method listed is relevant to your study. If you are not sure if a list item applies to your research, read the appropriate section before selecting a response.

Materials & experimental systems

n/a	Involved in the study	
[ ]	[x]	Antibodies
[ ]	[x]	Eukaryotic cell lines
[x]	[ ]	Palaeontology and archaeology
[x]	[ ]	Animals and other organisms
[x]	[ ]	Human research participants
[x]	[ ]	Clinical data
[x]	[ ]	Dual use research of concern

Methods

n/a	Involved in the study	
[x]	[ ]	ChIP-seq
[x]	[ ]	Flow cytometry
[x]	[ ]	MRI-based neuroimaging

Antibodies

Antibodies used

Anti-AHCTF1 (1:40 HPA031658, Sigma-Aldrich) .
Anti-rabbit antibody (1:150, 20183, Biotium, Fremont, CA).
Mouse anti-Nup-62 primary antibody (1:50, 610498, BD Bioscience).
Anti-HA (1:250, mouse monoclonal, 2-2.2.14, 26183,Thermo Fisher Scientific).
Anti-HIM-3 (1:250, rabbit polyclonal, 53470002, Novus Biologicals).
Anti-mouse-DY634 (1:150, 634-01, Dyomics).
Anti-HTP-3 (1:250, chicken polyclonal, gift from A. Dernburg).
Anti-rabbit IgG (1:100, donkey polyclonal, AB_2340586, Jackson Immunoresearch) .
Anti-chicken IgY (1:100, donkey polyclonal, AB_2340347, Jackson Immunoresearch) .
Anti-mouse IgG (1:100, donkey polyclonal, AB_2340761, Jackson Immunoresearch).
AlexaFluor 546 anti-mouse IgG (1:500, A10040, ThermoFischer Scientific).
AlexaFluor 647 anti-rabbit IgG (1:500, donkey polyclonal, 711-605-152, Jackson Immunoresearch).
AlexaFluor 488 anti-chicken IgY (1:500, donkey polyclonal, 703-545-155, Jackson Immunoresearch).

Validation

Validations were performed by the respectively indicated manufacturers/provider. These antibodies were used to create specimens for demonstrating a new fitting method for SMLM. The specificity in immunostaining serves as an internal validation.

Eukaryotic cell lines

Policy information about cell lines

Cell line source(s)	U2OS Nup96-SNAP-tag, catalog no. 300444, CLS Cell Line Service, Eppelheim, Germany.
Authentication	Cell lines were not further authenticated.
Mycoplasma contamination	Cells were tested negative for mycoplasma contamination.
Commonly misidentified lines (See ICLAC register)	No commonly misidentified cell lines were used.
