# Supplementary material for: Global fitting for high-accuracy multi-channel single-molecule localization
Source: Nat Commun. 2022 Jun 6;13:3133. doi: 10.1038/s41467-022-30719-4 (PMC9170706; doi:10.1038/s41467-022-30719-4)
Supplement: Supplementary file 5 — Supplementary Software [file 41467_2022_30719_MOESM5_ESM.zip › Supplementary Software 1/Installation guide for Supplementary Software.pdf]

Using globLoc in supplementary software

GlobLoc is designed as an independent module that can be integrated with custom software. We implemented the CUDA C/C++ source code as dynamic-link libraries which can be directly called from MATLAB and Python environments. For both MATLAB and Python, we provide 4 example codes for biplane, ratiometric, Gaussian and 4Pi single molecule localization as a template for using globLoc in own software.

Requirements

GlobLoc_matlab

- Matlab R2019a or newer
- Curve Fitting Toolbox
- Optimization Toolbox

The GPU fitter requires:

- Microsoft Windows 7 or newer, 64-bit
- CUDA capable graphics card, minimum Compute Capability 6.1
- CUDA 10.1 compatible graphics driver (for GeForce products 471.41 or later)

The CPU version runs on macOS and Microsoft Windows 7 or newer, 64-bit.

GlobLoc_python

Requires python 3.8 or newer. Installation instructions:

```
pip install numba
```

more details on https://numba.readthedocs.io/en/stable/user/installing.html

```
pip install tqdm
```

more details on https://github.com/tqdm/tqdm#installation

The GPU fitter requires:

- Microsoft Windows 7 or newer, 64-bit
- CUDA capable graphics card, minimum Compute Capability 6.1
- CUDA 10.1 compatible graphics driver (for GeForce products 471.41 or later)
- The CPU version runs on macOS and Microsoft Windows 7 or newer, 64-bit

How to run

GlobLoc_matlab

Examples code are avalible in file **Example_GlobalFit_4Pi.m, Example_GlobalFit_biplane.m, Example_GlobalFit_Gauss.m, Example_GlobalFit_Ratiometric.m**. The required test data for the demo code can be found at https://www.ebi.ac.uk/biostudies/studies/S-BSST839. GlobLoc has been fully integrated in fit3Dcspline plugin of SMAP (https://github.com/jries/SMAP).

GlobLoc_python

Examples code are avalible in file **example_4pi.py, example_biplane.py, example_gauss.py, example_ratiometric.py**.The required test data for the demo code can be here: https://www.ebi.ac.uk/biostudies/studies/S-BSST839. The results of the example can be found in the folder **output**.
