# Supplementary figures and images for: Global fitting for high-accuracy multi-channel single-molecule localization
Source: Nat Commun. 2022 Jun 6;13:3133. doi: 10.1038/s41467-022-30719-4 (PMC9170706; doi:10.1038/s41467-022-30719-4)

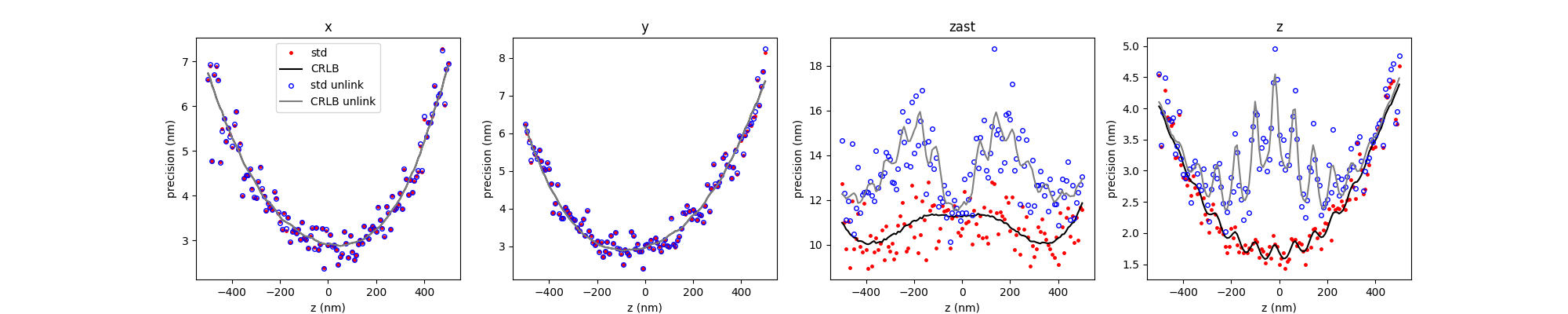

Supplement: Supplementary file 5 — Supplementary Software [file 41467_2022_30719_MOESM5_ESM.zip › Supplementary Software 1/GlobLoc/GlobLoc_python/output/output_4pi.png]

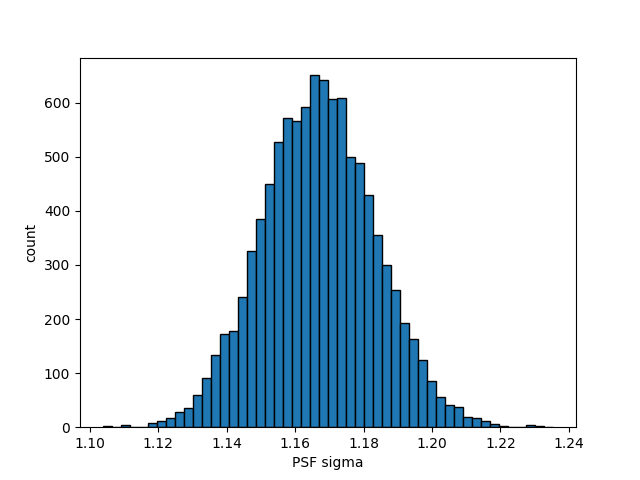

Supplement: Supplementary file 5 — Supplementary Software [file 41467_2022_30719_MOESM5_ESM.zip › Supplementary Software 1/GlobLoc/GlobLoc_python/output/output_psf_sigma.png]

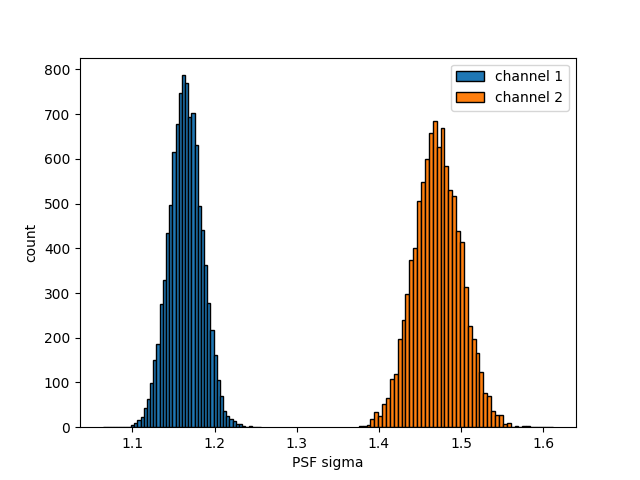

Supplement: Supplementary file 5 — Supplementary Software [file 41467_2022_30719_MOESM5_ESM.zip › Supplementary Software 1/GlobLoc/GlobLoc_python/output/output_psf_sigma_multi.png]

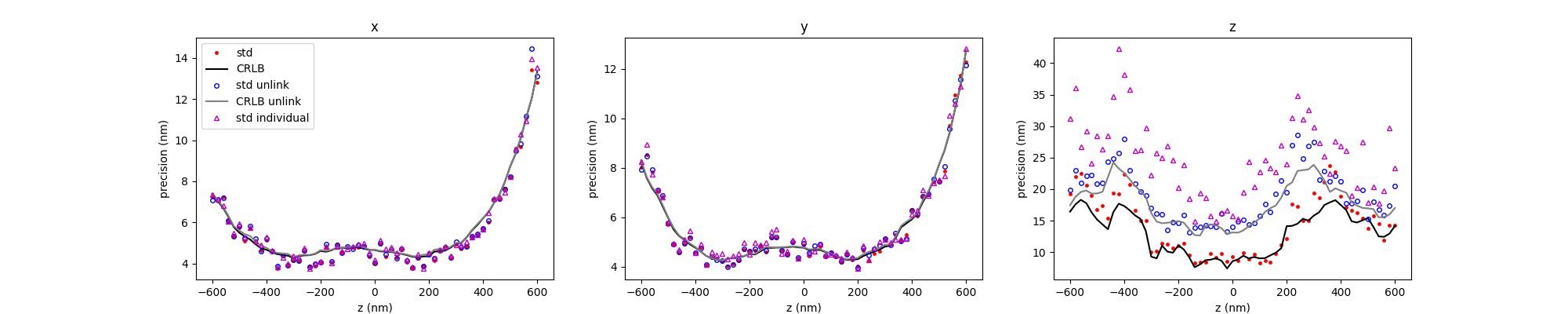

Supplement: Supplementary file 5 — Supplementary Software [file 41467_2022_30719_MOESM5_ESM.zip › Supplementary Software 1/GlobLoc/GlobLoc_python/output/output_biplane.png]

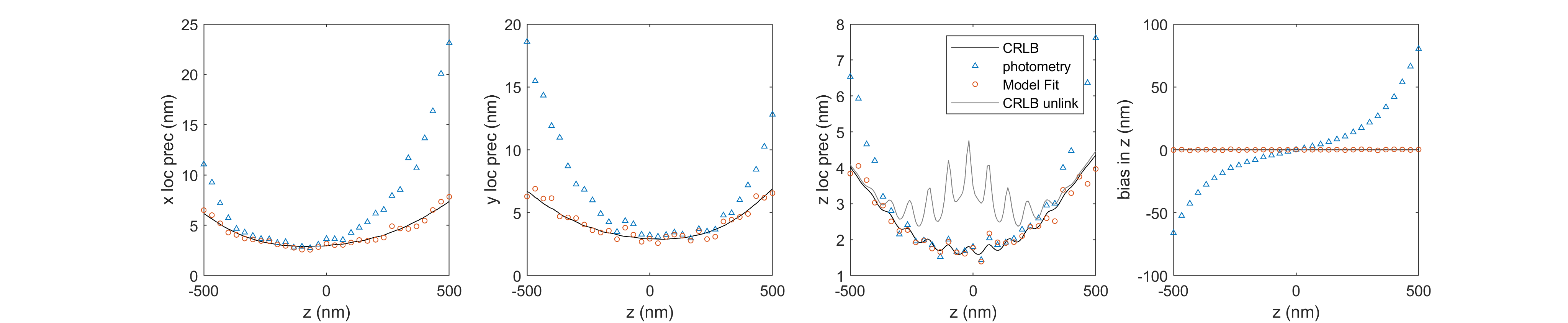

Supplement: Supplementary file 5 — Supplementary Software [file 41467_2022_30719_MOESM5_ESM.zip › Supplementary Software 1/GlobLoc/GlobLoc_matlab/private/output/std_crlb_vec.png]
