# Supplementary material for: Global fitting for high-accuracy multi-channel single-molecule localization
Source: Nat Commun. 2022 Jun 6;13:3133. doi: 10.1038/s41467-022-30719-4 (PMC9170706; doi:10.1038/s41467-022-30719-4)
Supplement: Supplementary file 6 — Description of Additional Supplementary Files [file 41467_2022_30719_MOESM6_ESM.pdf]

Supplementary Movie 1

Description: Average of 200 four color 3D super-resolution images of Nuclear pore complex

Supplementary Software 1

Description: GlobLoc is a graphics processing unit (GPU) based global fitting algorithm with flexible PSF modeling and parameter sharing, to extract maximum information from multi-channel single molecule data.
